# Supplementary material for: Low T cell diversity associates with poor outcome in bladder cancer: A comprehensive longitudinal analysis of the T cell receptor repertoire
Source: Cell Rep Med. 2025 May 1;6(5):102101. doi: 10.1016/j.xcrm.2025.102101 (PMC12147909; doi:10.1016/j.xcrm.2025.102101)
Supplement: Document S1. Figures S1–S7 and Tables S1–S2 [file mmc1.pdf]

Cell Reports Medicine, Volume *6*

## Supplemental information

# Low T cell diversity associates with poor outcome in bladder cancer: A comprehensive longitudinal analysis of the T cell receptor repertoire

**Asbjørn Kjær, Nanna Kristjánsdóttir, Randi Istrup Juul, Iver Nordentoft, Karin Birkenkamp-Demtröder, Johanne Ahrenfeldt, Trine Strandgaard, Deema Radif, Darren Hodgson, Christopher Abbosh, Hugo J.W.L. Aerts, Mads Agerbæk, Jørgen Bjerggaard Jensen, Nicolai J. Birkbak, and Lars Dyrskjøt**

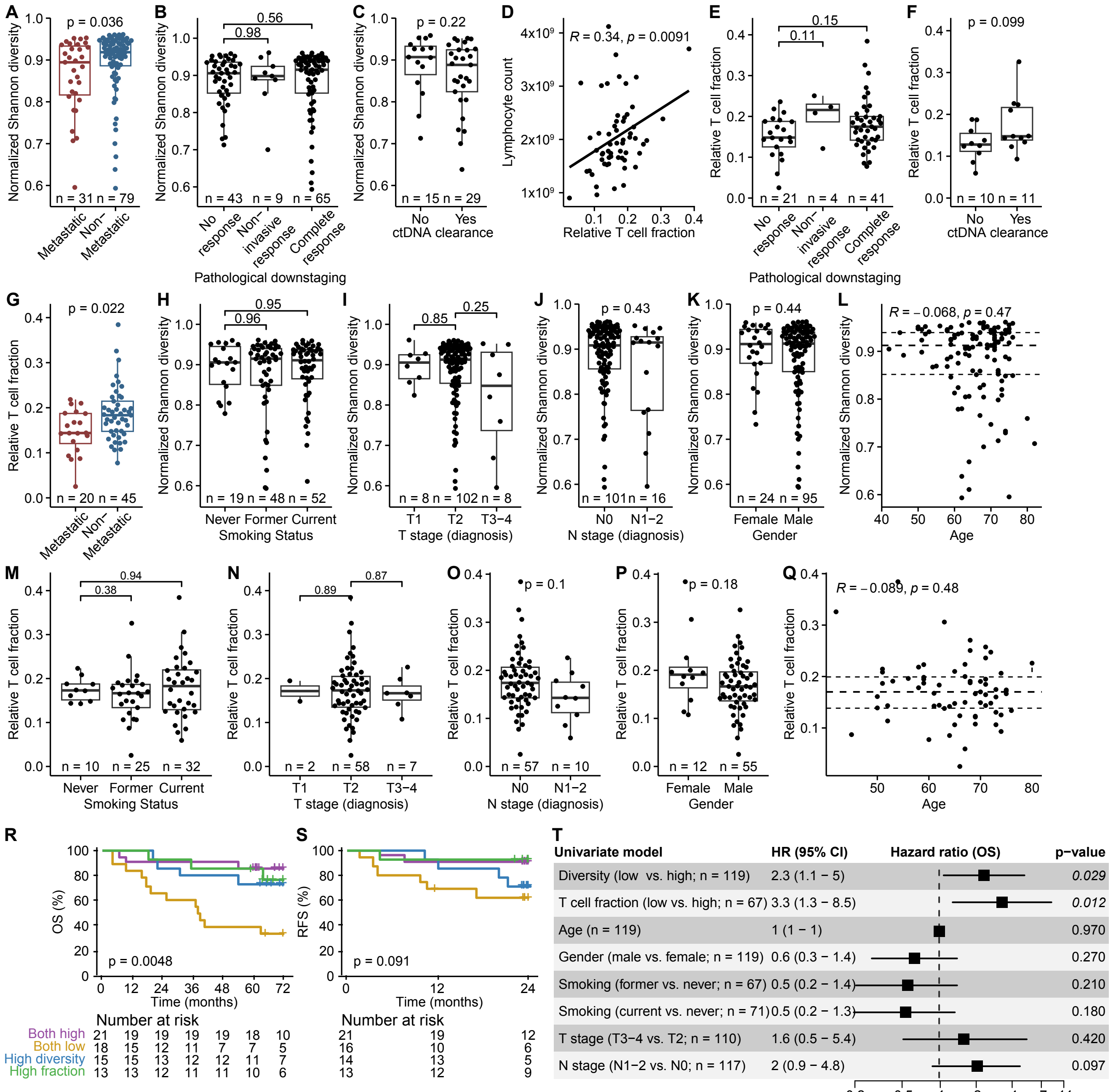


**Figure S1. Baseline peripheral TCR features, relative T cell fraction, and clinical parameters in MIBC, related to Figure 2.**
(A) Test of difference between normalized Shannon diversity and metastatic disease.
(B) Test of difference between normalized Shannon diversity and pathological downstaging.
(C) Test of difference between normalized Shannon diversity and ctDNA clearance.
(D) Spearman correlation of relative T cell fraction and lymphocyte count (n = 58).
(E) Test of difference between relative T cell fraction and pathological downstaging.
(F) Test of difference between relative T cell fraction and ctDNA clearance.
(G) Test of difference between relative T cell fraction and metastatic disease.
(H) Test of difference between normalized Shannon diversity and smoking status.
(I) Test of difference between normalized Shannon diversity and T stage.
(J) Test of difference between normalized Shannon diversity and N stage.
(K) Test of difference between normalized Shannon diversity and gender.
(L) Spearman correlation of normalized Shannon diversity and age (dashed lines indicate quartiles; n = 119)
(M) Test of difference between relative T cell fraction and smoking status.
(N) Test of difference between relative T cell fraction and T stage.
(O) Test of difference between relative T cell fraction and N stage.
(P) Test of difference between relative T cell fraction and gender.
(Q) Spearman correlation of relative T cell fraction and age (dashed lines indicate quartiles; n = 67).
(R) Survival analysis of OS for normalized Shannon diversity combined with relative T cell fraction, both split by median. High diversity indicates a high normalized Shannon diversity and low T cell fraction. High fraction indicates a high T cell fraction and low normalized Shannon diversity index.
(S) Survival analysis of RFS for normalized Shannon diversity combined with relative T cell fraction, both split by median. High diversity indicates a high normalized Shannon diversity and low T cell fraction. High fraction indicates a high T cell fraction and low normalized Shannon diversity index.
(T) Forest plot showing the HR for OS in univariate models of TCR diversity, T cell fraction, age, gender, smoking status, T stage, and N stage.
CI: confidence interval.

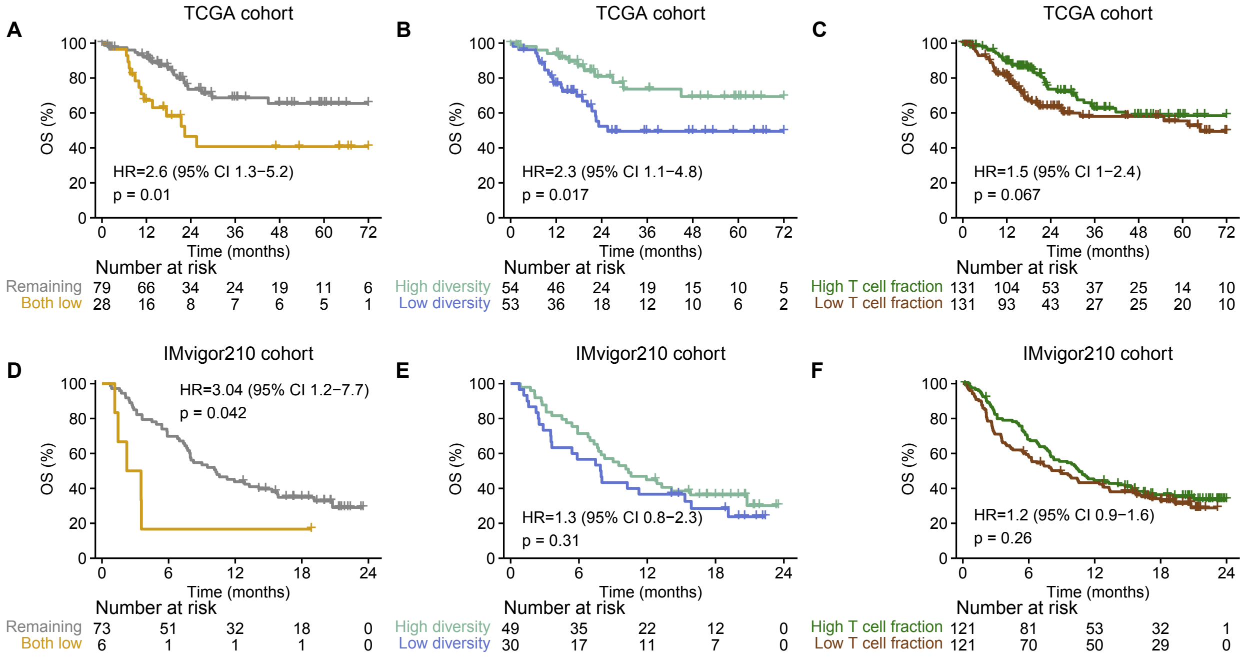


**Figure S2. Analysis of TCR diversity and T cell fraction in independent cohorts, related to Figure 2.**

(A-C) Survival analyses of stage I-III MIBC from TCGA (n = 262). The normalized Shannon diversity was determined by extracting TCR-beta sequences from germline WES samples. Only 107 patients had sufficient coverage of the CDR3 region to determine diversity. Survival analysis comparing OS for patients with low TCR diversity (median split) combined with low T cell fraction (median split) against the remaining patients (A), low and high (median split) TCR diversity (B), and low and high (median split) T cell fraction (C).

(D-F) Survival analyses of patients with metastatic bladder cancer from IMvigor210 (n = 242, n diversity = 79). Survival analysis comparing OS for patients with low TCR diversity (median split) combined with low T cell fraction (median split) against the remaining patients (D), low and high (median split) TCR diversity (E), and low and high (median split) T cell fraction (F).

CI: confidence interval.

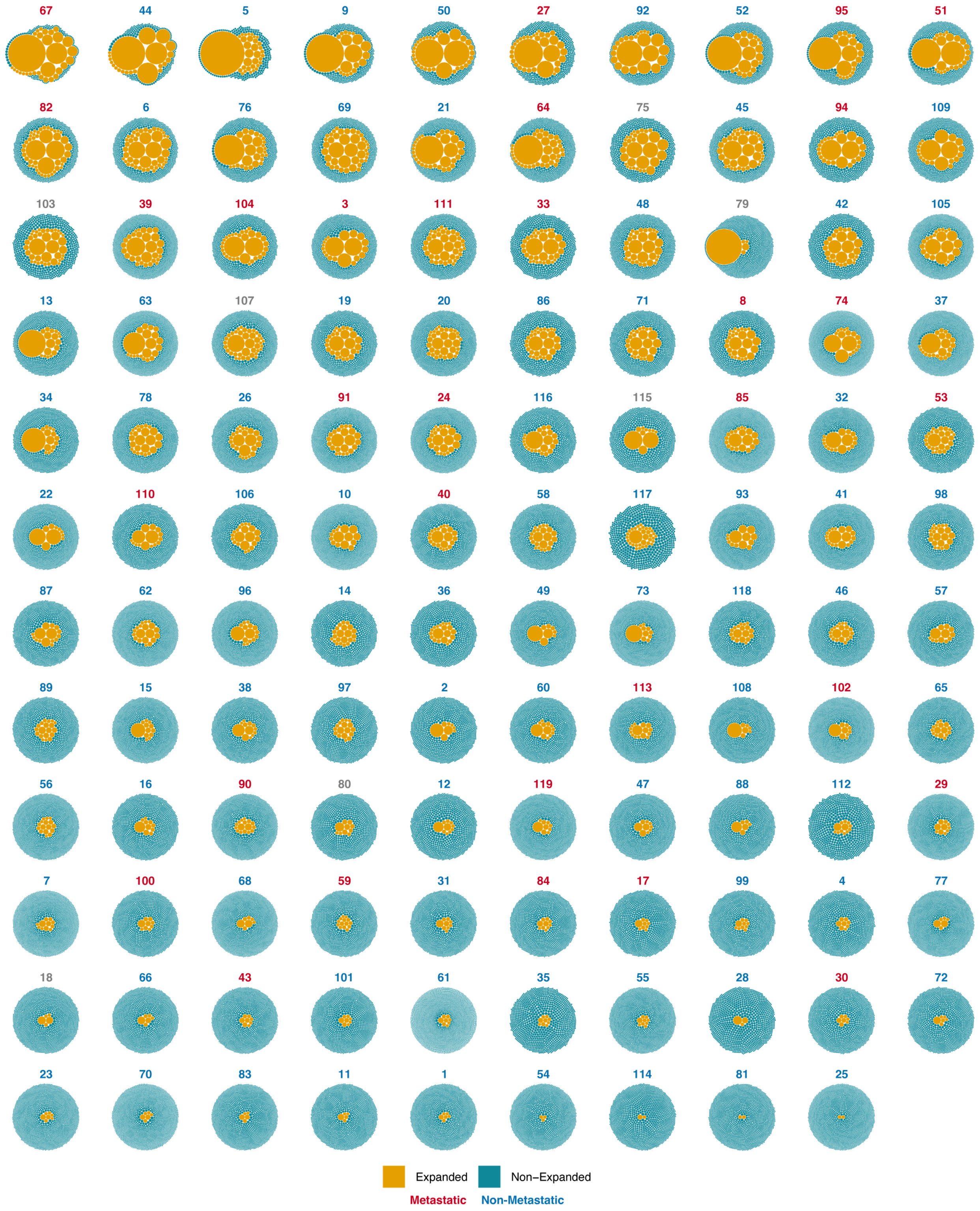


**Figure S3. Bubble plots of baseline blood samples from the MIBC cohort, related to Figure 3.**
One plot per patient. Each bubble represents a single TCR clone, with the size of the bubble representing the clone size. Colored by the size threshold for expansion. Patient labels colored by later development of metastatic disease.

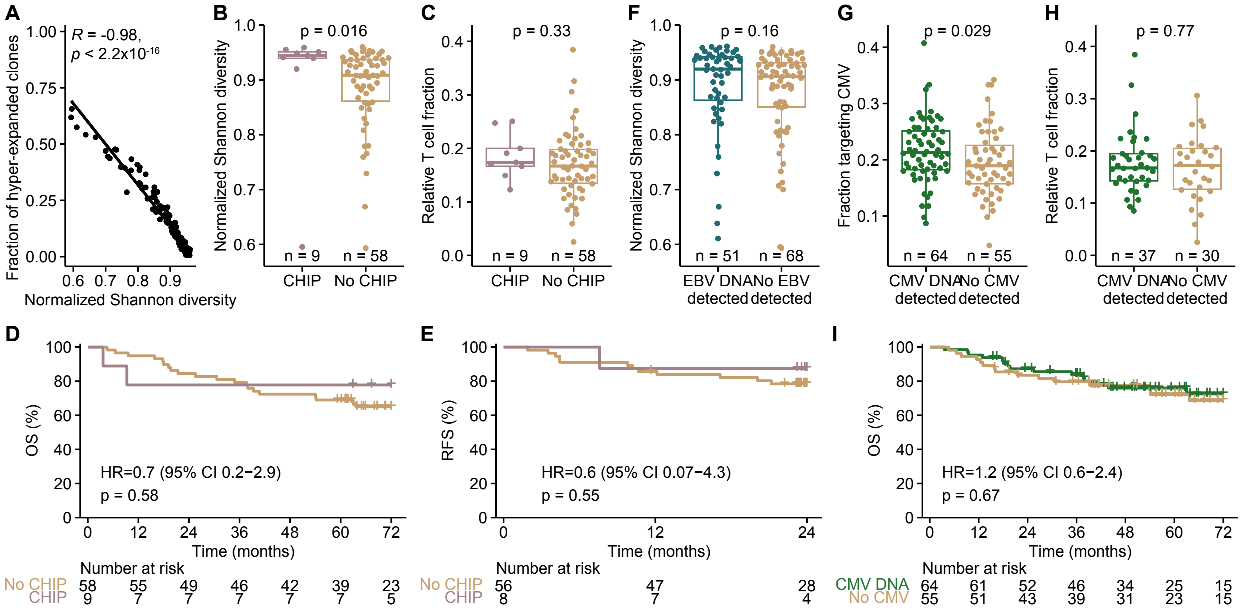

**Figure S4. Analyses of CHIP and latent viral infections, related to Figure 3.**

(A) Spearman correlation between the fraction of hyper-expanded clones and normalized Shannon diversity (n = 119).

(B-E) Analysis of CHIP in patients with MIBC. CHIP was called based on the detection of somatic, CHIP-associated mutations found in germline WES data.

(B) Test of difference between normalized Shannon diversity and CHIP.

(C) Test of difference between relative T cell fraction and CHIP.

(D) Survival analysis of OS for patients with and without detectable CHIP.

(E) Survival analysis of RFS for patients with and without detectable CHIP.

(F-I) Detection of CMV and EBV DNA in patients with MIBC. CMV and EBV DNA were detected based on WGS data from plasma samples using Kraken2.

(F) Test of difference between normalized Shannon diversity and detection of EBV DNA.

(G) Test of difference between fraction of TCRs targeting CMV and detection of CMV DNA.

(H) Test of difference between relative T cell fraction and detection of CMV DNA.

(I) Survival analysis of OS for patients with and without detectable CMV DNA.

CI: confidence interval.

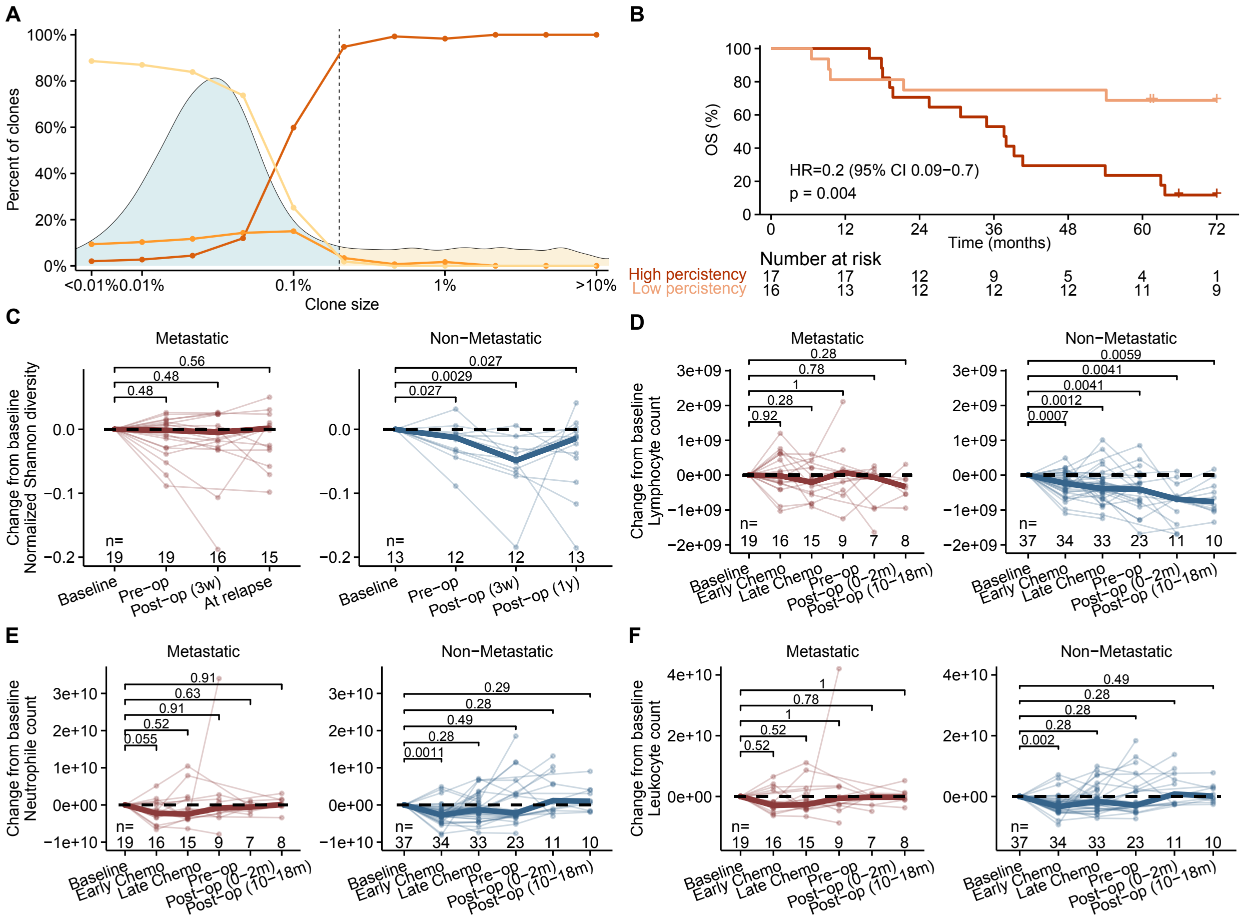


**Figure S5. Analysis of persistency and blood lab measurements in MIBC, related to Figure 4.**

(A) Association between clonal persistence and size of clones. Lines indicate the percentage of clones in each category at a specific size bin. Bins are uniformly distributed on log scale, x-axis placement indicates the end of the bin. All clones were concatenated for this analysis. The density plot shows the baseline size distribution of T cell clones (from Figure 3A). The dashed line as well as the blue and yellow colors indicate the threshold for defined hyper-expanded T cell clones.

(B) Survival analysis showing that high persistency (above median amounts of persistent and recurrent clones) is associated with OS.

(C-F) Change in immune landscape throughout treatment for patients with and without metastatic disease separately (two patients excluded due to incomplete follow-up); normalized Shannon diversity (C), lymphocyte counts (D), neutrophil counts (E), and leukocyte counts (F). Early chemo represents samples taken within one month of initiation, and late chemo represents the remaining samples. When multiple samples were available the average count was used. The thick lines indicate the median. Tests are based on the raw diversity measures or lab counts. P-values are FDR-adjusted.

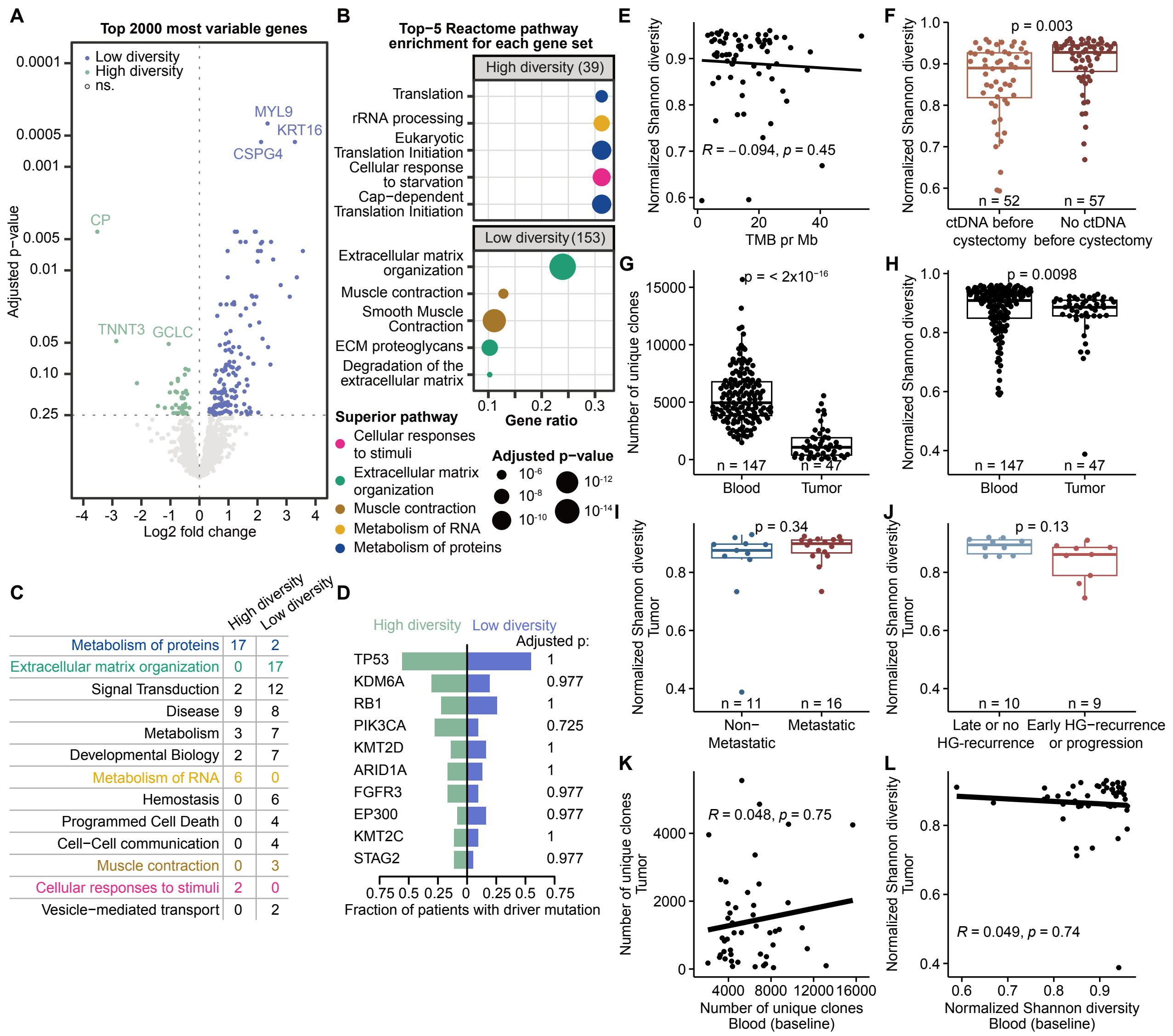


**Figure S6. Association of peripheral TCR repertoires and tumor biology or tumor TCR repertoires, related to Figure 6.**

(A) Differential gene expression analysis of the top 2000 most variable genes comparing patients with high and low TCR diversity for patients with MIBC (low diversity, n = 23; high diversity, n = 21).

(B) Reactome pathway enrichment analysis using significantly differentially expressed genes with higher expression in patients with high (n = 39) and low (n = 153) TCR diversity. The top five pathways for each group are shown.

(C-F) Analysis of peripheral TCR diversity relative to tumor biology.

(C) Summary of significant pathways from the Reactome pathway analysis in Figure 6B.

(D) Tree-plot showing the difference in the number of driver mutations between patients with high and low TCR diversity in ten bladder cancer driver genes (n, high diversity = 36; n, low diversity = 31).

(E) Spearman correlation of normalized Shannon diversity and TMB (n = 67).

(F) Test of difference between normalized Shannon diversity and ctDNA detection for patients with and without detectable levels of ctDNA before cystectomy.

(G-L) Joint analysis of paired tumor and baseline repertoires for patients with MIBC (n, blood = 119; n, tumor = 28) and NMIBC (n, blood = 28; n, tumor = 19).

(G) Test of difference between the number of unique clones in blood and tumor.

(H) Test of difference between the normalized Shannon diversity in blood and tumor.

(I) Test of difference between normalized Shannon diversity in the tumor and development of metastatic disease for patients with MIBC, with and without metastasis.

(J) Test of difference between normalized Shannon diversity in the tumor and recurrence status for patients with NMIBC, with late or no HG recurrence and early HG recurrence or progression.

(K) Spearman correlation between the number of unique clones in the tumor and in the blood (n = 47).

(L) Spearman correlation between the normalized Shannon diversity in the tumor and in the blood (n = 47).

HG: high-grade. TMB: tumor mutation burden.

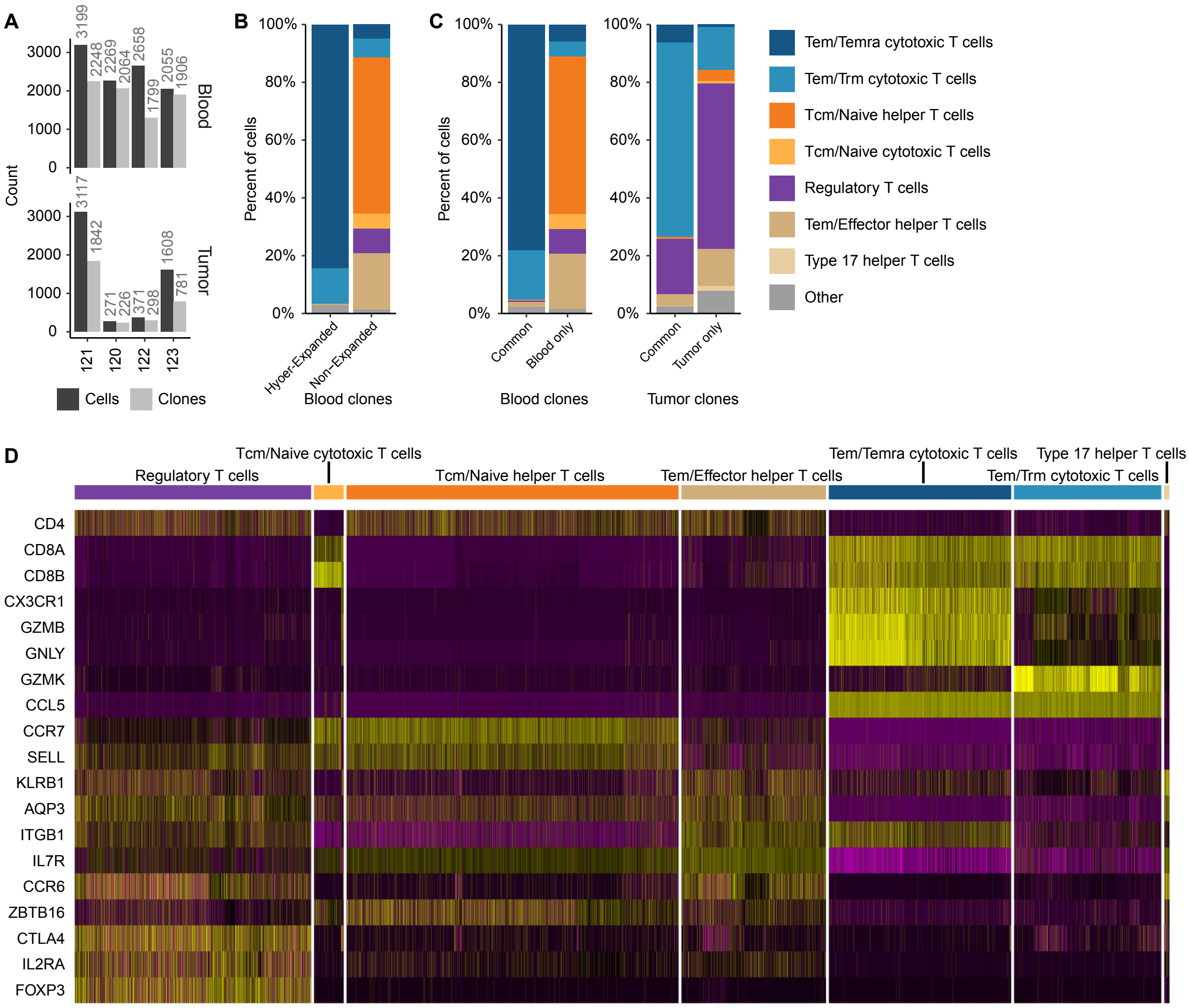


**Figure S7. Single-cell analyses, related to Figure 7.**

(A) Bar plots showing the number of T cells and unique clones for blood and tumor.

(B) Bar plots showing the distribution of cell types split by the T cell receptor being categorized as a hyper-expanded or non-expanded clone (n, cells with hyper-expanded clone = 2312; n, cells with non-expanded clone = 7869).

(C) Bar plots showing the distribution of cell types in blood and tumor cells, respectively, split by the TCR being either a common or a unique clone (n, cells with common clone in blood = 2390; n, cells with unique clone in blood = 7791; n, cells with common clone in tumor = 727; n, cells with unique clone in tumor = 4640).

(D) Heatmap showing gene expression from curated marked genes.

Tem: effector memory T cell. Temra: effector memory T cell reexpressing CD45RA. Trm: tissue-resident memory T cell. Tcm: central memory T cell.

**Table S1 | Overview of analyzed samples, related to Figure 1**

## MIBC cohort (n = 119)

*Patients from a prospective study cohort*

*Inclusion criteria: Neoadjuvant chemotherapy preceding radical cystectomy and available baseline blood sample*

| Data produced for this study | | Previously produced data | |
|---|---|---|---|
| TCRseq | Baseline blood samples (n = 119) | WGS | Plasma samples (n = 119) |
| | Longitudinal blood samples (multiple time points, n = 33 patients) | WES | Blood and tumor samples (n = 67) |
| | Tumor samples (n = 30) | RNAseq | Tumor samples (n = 44) |
| RNAseq | Baseline blood samples (n = 8) | | |

**Available laboratory blood measurements**

Baseline and longitudinal measurements (multiple time points, n = 58 patients)

## NMIBC cohort (n = 30)

*Subset of patients from a retrospective study*

*Inclusion criteria: Treated with a minimum of 5 BCG instillations*

| Data produced for this study | | Previously produced data (extended cohort) | |
|---|---|---|---|
| TCRseq | Baseline blood samples (n = 28) | WES | Blood and tumor samples (n = 110) |
| | After BCG blood samples (n = 28) | | |
| | Tumor samples (n = 19) | | |

## Single-cell cohort (n = 4)

*New patients included for this study*

*Inclusion criteria: Chemotherapy-naive patients with bladder cancer undergoing a radical*

| Data produced for this study | | | |
|---|---|---|---|
| scRNAseq | Baseline blood samples (n = 4) | | |
| | Tumor samples (n = 4) | | |

scRNAseq: single-cell RNAseq

**Table S2 | Summary of patient characteristics, related to Figure 1**

| | MIBC (n = 119) | NMIBC (n = 30) |
|---|---|---|
| **Gender** | | |
| Female | 24 (20.2%) | 7 (23.3%) |
| Male | 95 (79.8%) | 23 (76.7%) |
| **Age*** | | |
| Mean (SD) | 70 (8) | 70 (10) |
| Median [min, max] | 70 [40, 80] | 70 [50, 80] |
| **Smoking status** | | |
| Current | 52 (43.7%) | 17 (56.6%) |
| Former | 48 (40.3%) | 11 (36.7%) |
| Never | 19 (16.0%) | 2 (6.7%) |
| **T stage at diagnosis** | | |
| Ta | 0 (0%) | 18 (60.0%) |
| T1 | 8 (6.7%) | 12 (40.0%) |
| T2 | 102 (85.8%) | 0 (0%) |
| T3 | 1 (0.8%) | 0 (0%) |
| T4a | 4 (3.4%) | 0 (0%) |
| T4b | 3 (2.5%) | 0 (0%) |
| Tx | 1 (0.8%) | 0 (0%) |
| **N stage at diagnosis** | | |
| N0 | 101 (84.9%) | 30 (100%) |
| N1 | 13 (10.9%) | 0 (0%) |
| N2 | 3 (2.5%) | 0 (0%) |
| Missing | 2 (1.7%) | 0 (0%) |
| **Recurrence**** | | |
| Recurrence | 31 (26.0%) | 15 (50.0%) |
| No recurrence | 79 (66.4%) | 15 (50.0%) |
| Follow-up < two years | 9 (7.6%) | 0 (0%) |

* MIBC: age at diagnosis; NMIBC: age at BCG induction

** MIBC: detection of metastasis; NMIBC: early high-grade recurrence (< two years) or progression

SD: standard deviation
