# Supplementary material for: Low T cell diversity associates with poor outcome in bladder cancer: A comprehensive longitudinal analysis of the T cell receptor repertoire
Source: Cell Rep Med. 2025 May 1;6(5):102101. doi: 10.1016/j.xcrm.2025.102101 (PMC12147909; doi:10.1016/j.xcrm.2025.102101)
Supplement: Document S2. Article plus supplemental information [file mmc5.pdf]

Cell Reports
Medicine

Article

Low T cell diversity associates with poor outcome in bladder cancer: A comprehensive longitudinal analysis of the T cell receptor repertoire

Graphical abstract

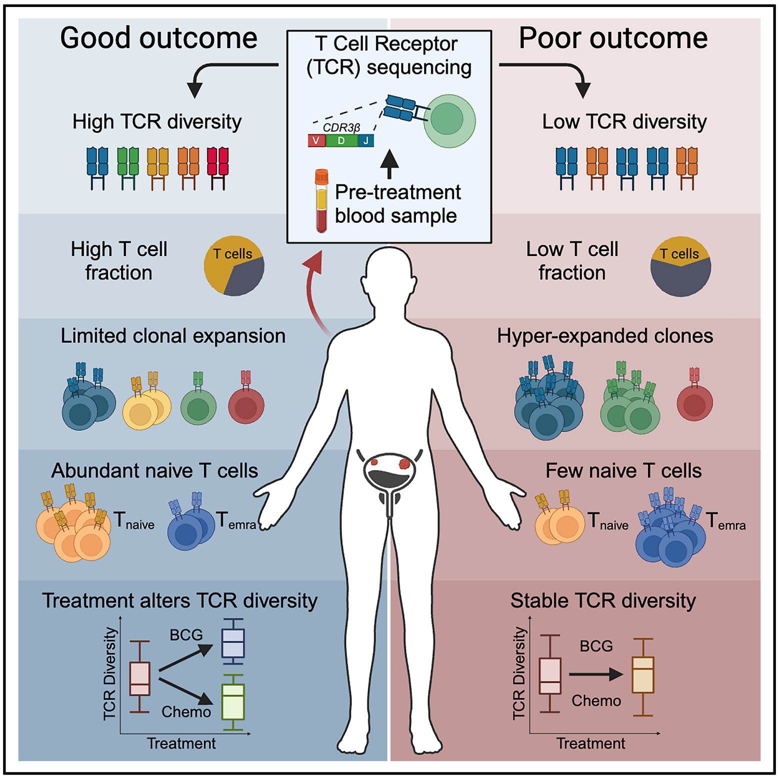

Highlights

- Low peripheral TCR diversity and T cell fraction are associated with worse outcomes
- Low peripheral TCR diversity is associated with persistent hyper-expanded clones
- Hyper-expanded clones are primarily terminally differentiated cytotoxic T cells
- Oncological treatment affects TCR diversity in patients with good outcomes

Authors

Asbjørn Kjær, Nanna Kristjánsdóttir, Randi Istrup Juul, ..., Jørgen Bjerggaard Jensen, Nicolai J. Birkbak, Lars Dyrskjøt

Correspondence

nbirkbak@clin.au.dk (N.J.B.),
lars@clin.au.dk (L.D.)

In brief

Kjær et al. comprehensively characterize the TCR repertoire in bladder cancer, finding that low peripheral TCR diversity and low T cell fraction are associated with poor prognosis. Low diversity is correlated with persistent hyper-expanded clones. Longitudinal analysis reveals that treatment changes TCR diversity in patients with favorable outcomes.

Kjær et al., 2025, Cell Reports Medicine 6, 102101
May 20, 2025 © 2025 The Author(s). Published by Elsevier Inc.
https://doi.org/10.1016/j.xcrm.2025.102101

CellPress

Cell Reports Medicine

CellPress OPEN ACCESS

Article

Low T cell diversity associates with poor outcome in bladder cancer: A comprehensive longitudinal analysis of the T cell receptor repertoire

Asbjørn Kjær,[1,2,8] Nanna Kristjánsdóttir,[1,2,8] Randi Istrup Juul,[1,2,8] Iver Nordentoft,[1] Karin Birkenkamp-Demtröder,[1,2] Johanne Ahrenfeldt,[1] Trine Strandgaard,[1,2] Deema Radif,[1,2] Darren Hodgson,[3] Christopher Abbosh,[3] Hugo J.W.L. Aerts,[4,5] Mads Agerbæk,[6] Jørgen Bjerggaard Jensen,[2,7] Nicolai J. Birkbak,[1,2,9,10,*] and Lars Dyrskjøt[1,2,9,10,11,*]

[1]Department of Molecular Medicine, Aarhus University Hospital, 8200 Aarhus N, Aarhus, Denmark
[2]Department of Clinical Medicine, Aarhus University, 8000 Aarhus, Denmark
[3]Cancer Biomarker Development, Oncology R&D, AstraZeneca, Cambridge CB2 0AA, UK
[4]Artificial Intelligence in Medicine (AIM) Program, Mass General Brigham, Harvard Medical School, Boston, MA 02114, USA
[5]Radiology and Nuclear Medicine, CARIM & GROW, Maastricht University, 6200 MD Maastricht, the Netherlands
[6]Department of Oncology, Aarhus University Hospital, 8200 Aarhus N, Aarhus, Denmark
[7]Department of Urology, Aarhus University Hospital, 8200 Aarhus N, Aarhus, Denmark
[8]These authors contributed equally
[9]These authors contributed equally
[10]Senior authors
[11]Lead contact
*Correspondence: nbirkbak@clin.au.dk (N.J.B.), lars@clin.au.dk (L.D.)
https://doi.org/10.1016/j.xcrm.2025.102101

SUMMARY

T cells are crucial effector cells in the endogenous defense against cancer, yet the clinical impact of their quantity, diversity, and dynamics remains underexplored. Here, we investigate the clinical relevance of the T cell receptor (TCR) repertoire in patients with bladder cancer. In advanced-stage disease, low pre-treatment peripheral TCR diversity is associated with worse overall survival ($p = 0.024$), particularly when coupled with low circulating T cell fractions ($p = 0.00049$). These low-diversity repertoires are dominated by hyper-expanded clones that persist throughout treatment. Further longitudinal analysis reveals reductions in TCR diversity after treatment, indicating adverse effects on the immune system. In early-stage disease, immunotherapy increases TCR diversity in patients with good outcomes. Furthermore, single-cell sequencing identifies most hyper-expanded clones as cytotoxic T cells, while non-expanded clones are predominantly naive T cells. Overall, this highlights TCR diversity as a promising biomarker, offering opportunities for tailored oncological treatments to enhance clinical outcomes.

INTRODUCTION

Cancer is generally considered a disease primarily driven by somatic alterations in the genome. However, there is now increasing evidence suggesting that cancer development and progression may also be promoted by a dysfunctional immune response,[1–3] likely shaping cancer evolution through selection for immune-resistant cancer clones. T cells are critical components of the adaptive immune system and central to the endogenous anti-tumor response. T cells carry unique T cell receptors (TCRs) generated by DNA rearrangements as they mature in the thymus. These receptors enable T cells to provide a tailored and memory-based defense by recognizing foreign antigens on the surface of antigen-presenting cells with high specificity. Proliferation following T cell activation results in the expansion of T cell clones sharing identical TCRs. This mechanism of action enables the evaluation of the T cell clone landscape through analysis of the TCR repertoire. Quantifying the TCR repertoire in circulation can reveal the breadth of a potential T cell response through analysis of TCR diversity and the strength of an ongoing response through evaluation of clonal expansion. While most TCR targets remain unknown and may be unrelated to cancer, the TCR repertoire itself can provide insights into the current state of the immune system, which may affect patient outcomes.

Bladder cancer is a highly immunogenic disease characterized by one of the highest tumor mutation burdens,[4] strong immune cell infiltration of the tumor microenvironment, and the formation of tertiary lymphoid structures within the tumor periphery. Treatment is stratified by cancer invasiveness. In high-risk non-muscle-invasive bladder cancer (NMIBC), immunotherapy based on Bacillus Calmette-Guerin (BCG) instillations is the standard of care and is highly effective in preventing disease recurrence and progression.[5] Platinum-based neoadjuvant chemotherapy followed by radical cystectomy is the preferred

© 2025 The Author(s). Published by Elsevier Inc.
This is an open access article under the CC BY license (http://creativecommons.org/licenses/by/4.0/).

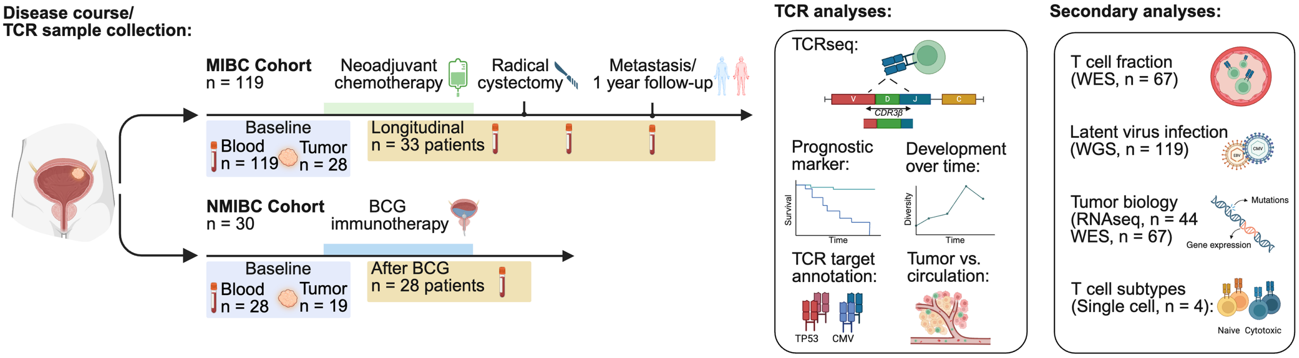

Figure 1. Study overview

Overview of the patient cohorts and study analyses. Blood vials indicate the time points for the collection of samples. Created with BioRender.com. See also Tables S1 and S2.

treatment for patients with localized muscle-invasive bladder cancer (MIBC). The treatment has high perioperative morbidity and mortality, and metastatic relapse is observed in about 50% of patients.[6] For patients not eligible for chemotherapy, immunotherapy is recommended as first-line treatment.[7] Additionally, recent studies have shown that combining immunotherapy and antibody-drug conjugate (pembrolizumab and enfortumab vedotin) significantly improves outcomes compared with standard chemotherapy.[8] As bladder cancer is highly immunogenic, investigating the TCR repertoire may significantly improve our understanding of the host anti-tumor response. Previous work has found the peripheral TCR repertoire to be associated with patient outcomes in multiple other cancer types,[9–15] indicating that the TCR repertoire affects tumor progression and may facilitate patient risk stratification. However, the impact of the peripheral TCR repertoire remains largely unexplored in bladder cancer. Additionally, the dynamics of the peripheral TCR repertoire during treatment and the interplay between tumor biology and the TCR repertoire remain underexplored.

Here, we present an in-depth analysis of the TCR repertoire in circulation to further our understanding of its biological impact and to investigate its potential for predicting clinical outcomes in patients with bladder cancer. Utilizing TCR sequencing (TCR-seq), we observe that patients with low TCR diversity have significantly shorter survival. These patients often harbor large hyper-expanded T cell clones that specifically target persistent viral infections. In a single-cell analysis, we find that these large hyper-expanded T cell clones are predominantly terminally differentiated cytotoxic T cells. We also find that TCR diversity is associated with distinct tumor biology, indicating that the T cell landscape may affect cancer development. Furthermore, through longitudinal analysis of TCR repertoire dynamics, we demonstrate how treatment negatively impacts both short- and long-term TCR diversity and lymphocyte abundance, particularly among patients with good outcomes.

RESULTS

Patients, biological samples, and molecular data

To explore the T cell landscape, we performed TCR-seq on tumor biopsies and longitudinal blood samples from patients with MIBC (n = 119) and NMIBC (n = 30). This was analyzed together with whole-exome sequencing (WES), whole-genome sequencing (WGS), and transcriptome (RNA sequencing [RNA-seq]) sequencing data available from previous studies[1,16–19] (Figure 1; Table S1). Patients were treated and followed according to standard clinical guidelines at Aarhus University Hospital, Denmark. A summary of clinical and histopathological characteristics is provided in Table S2.

Low TCR diversity and low T cell fraction at baseline are associated with worse disease outcomes in MIBC

We investigated the peripheral blood TCR repertoire before chemotherapy (baseline) in 119 patients with MIBC using TCR-seq on buffy coat DNA. This resulted in a median recovery of 4,908 unique TCR CDR3β chains (hereafter referred to as T cell clones) per sample (range 1,479–15,679). To investigate the clinical impact of peripheral TCR diversity in MIBC, we determined the normalized Shannon diversity index, which quantifies the evenness of clones in a repertoire. We found that baseline TCR diversity was significantly associated with survival, where patients with below-median TCR diversity experienced significantly shorter overall survival (OS; hazard ratio [HR] = 2.3, p = 0.024; Figure 2A). They also had shorter recurrence-free survival (RFS), although it did not reach statistical significance (HR = 1.7, p = 0.22; Figure 2B). Patients who developed metastatic disease had significantly lower TCR diversity compared with those who did not develop metastases (p = 0.036; Figure S1A), although no association was found between baseline TCR diversity and chemotherapy efficacy (pathologic downstaging and circulating tumor DNA [ctDNA] clearance; Figures S1B and S1C).

TCR diversity measures the evenness of T cell clones but contains no information about the amount of T cells in circulation. To investigate the associations between TCR diversity, T cell levels, and outcomes, we determined the relative fraction of T cells in circulation before treatment in 67 patients with MIBC using TcellExTRECT[20] on germline buffy coat WES data. This measure of T cell fraction correlated with lymphocyte counts (Figure S1D). Patients with a below-median T cell fraction had a significantly shorter OS (HR = 3.3, p = 0.0075; Figure 2C) and RFS (HR = 4.2, p = 0.016; Figure 2D). As shown for TCR diversity,

CellPress
OPEN ACCESS

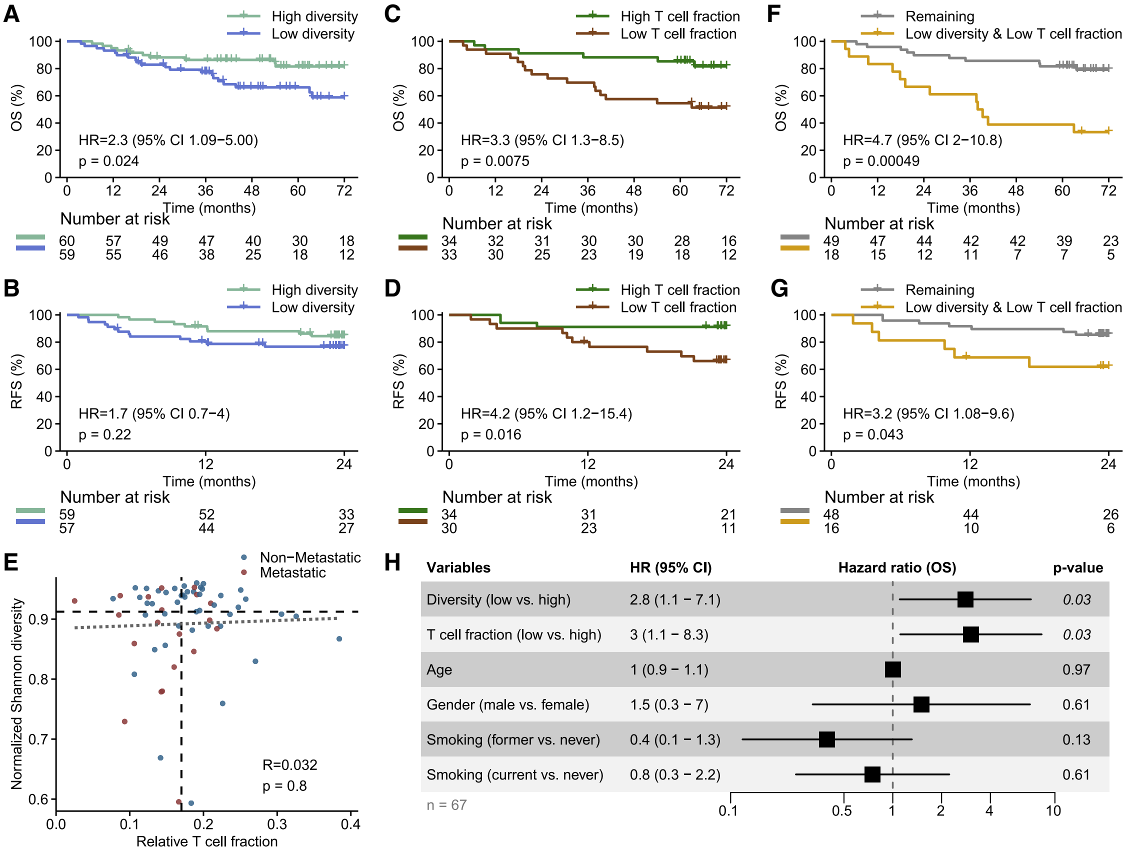

Figure 2. Baseline TCR diversity and T cell fraction are associated with overall survival

(A and B) Survival analysis associating TCR diversity (median split) with OS and RFS.

(C and D) Survival analysis associating T cell fraction (median split) with OS and RFS.

(E) Spearman correlation between normalized Shannon diversity and relative T cell fraction (*n* = 67). The dotted line shows a linear fit. Dashed lines show the medians.

(F and G) Survival analyses of OS and RFS comparing patients with low normalized Shannon diversity (below median) and low relative T cell fraction (below median) to all other patients.

(H) Forest plot showing the HR for OS in a multivariable analysis including TCR diversity, T cell fraction, age, gender, and smoking status. CI, confidence interval.
See also Figures S1 and S2.

T cell fraction was not associated with chemotherapy efficacy (Figures S1E and S1F), yet patients who developed metastases had a significantly lower fraction of T cells compared to patients who did not ($p = 0.022$; Figure S1G).

Interestingly, TCR diversity and T cell fraction were uncorrelated (Spearman's rho = 0.032, $p = 0.8$; Figure 2E), indicating that they represent independent measures of the T cell repertoire. Neither measure was associated with patient characteristics (Figures S1H–S1Q), although most patients in the lowest TCR diversity quartile were older than 60 years (28/30 patients, $p = 0.02$; Figure S1L). In a combined analysis, we found that patients with both below-median TCR diversity and below-median T cell fraction had shorter OS (HR = 4.7, $p = 0.00049$; Figures 2F and S1R) and shorter RFS (HR = 3.2, $p = 0.043$; Figures 2G and S1S) compared to the remaining patients. TCR diversity and relative T cell fraction were identified as the only significant predictors of OS in both univariate and multivariable analyses (Figures 2H and S1T). Together, these results indicate that peripheral blood TCR diversity and T cell fraction are both independently associated with outcomes for patients with MIBC and that the combination of low TCR diversity and low T cell fraction represents a particularly poor prognosis.

Lastly, we aimed to validate these findings in an independent cohort of patients with non-metastatic MIBC (stage I–III patients from The Cancer Genome Atlas [TCGA] cohort).[21] As deep TCR-seq data were unavailable, we used an orthogonal approach to determine TCR diversity. We utilized germline WES data to determine both peripheral TCR diversity and T cell fraction. Of 262 samples, 107 had sufficient TCR sequences to estimate TCR diversity. We observed that low TCR diversity and low T cell fraction were associated with shorter OS both combined and individually, analogous to the main cohort ($p = 0.01$, $p = 0.017$, $p = 0.067$; Figures S2A–S2C). In the same manner, we determined TCR diversity and T cell fraction in a cohort of patients treated with immunotherapy for metastatic bladder cancer (IMvigor210 cohort).[22] We again observed that patients with both low TCR diversity and low T cell fraction had significantly shorter OS (HR = 3.0, $p = 0.042$; Figure S2D), although neither low TCR

diversity nor low T cell fraction was associated with outcome individually (Figures S2E and S2F).

TCR clonal expansion varied substantially among patients with MIBC prior to chemotherapy

To further explore the TCR landscape, we determined the clone size distribution across all patients. This analysis revealed a right-skewed distribution composed of two distinct categories of T cell clones: small non-expanded T cell clones, each clone represented by few T cells, and large hyper-expanded T cell clones, each clone represented by many T cells (Figure 3A). Using a previously defined threshold,[23] we categorized the T cell population into hyper-expanded clones, represented at a frequency exceeding 0.2%, and non-expanded clones, represented at a frequency below 0.2%. The group of hyper-expanded clones constituted only 0.34% of the total amount of unique T cell clones while representing 17.25% of the total fraction of T cells (Figure 3B). We observed a considerable variation in clonal expansion across patients (with 0.5%–65% of T cells represented by hyper-expanded clones), corresponding to individual repertoires ranging from almost no clonal expansion to repertoires dominated by hyper-expanded T cell clones (Figures 3C and S3). Hyper-expanded clones skew the size distribution of T cell clones, causing a decrease in TCR diversity. Consequently, TCR diversity is strongly negatively correlated with the fraction of hyper-expanded T cell clones ($r = -0.98$, $p < 0.0001$; Figure S4A). This indicates that low TCR diversity is primarily driven by increased amounts of hyper-expanded clones.

Clonal hematopoiesis of indeterminate potential does not induce low TCR diversity

Clonal hematopoiesis of indeterminate potential (CHIP) is characterized by the clonal expansion of hematopoietic cells, induced by proliferative mutations. If a CHIP driving mutation occurs in a T cell, it could lead to the formation of an expanded T cell clone. To investigate if the observed T cell clonal expansion might be induced by lymphoid CHIP, we determined the prevalence of CHIP-associated somatic mutations in patients using germline WES data. We defined CHIP as previously described by Bick et al.,[24] requiring at least one CHIP-associated mutation observed at a minimum frequency of 2%. We detected CHIP in 13% (9/67) of patients with MIBC. These patients had significantly higher baseline TCR diversity than those without CHIP ($p = 0.016$; Figure S4B), indicating that CHIP does not promote clonal expansion of T cells. CHIP was neither associated with T cell fraction nor clinical outcomes (Figures S4C–S4E).

Hyper-expanded T cell clones primarily target antigens from latent viral infections

Next, we explored the antigen targets of the T cell clones from the baseline MIBC samples. First, we established clusters of highly homologous TCR sequences based on sequence similarity, using GLIPH2.[25] Likely antigen targets of these clusters were then inferred based on sequence similarity with known CDR3β-antigen pairs[26] (Figure 3D). This inference is limited by the availability of TCR-antigens pairs, and as this inference is based solely on the β chain, both false-positive targets and missing targets are expected. Using this method, we were only able to infer a likely target for a minority of the T cell clones. We found that the TCRs of hyper-expanded clones were more likely to have an inferred target relative to TCRs of non-expanded clones (2.2% vs. 0.9%; odds ratio = 2.55, $p = 3 \times 10^{-8}$; Figure 3E). We observed a marked difference between the inferred antigen targets of hyper-expanded and non-expanded clones (Figure 3F), with hyper-expanded clones significantly more likely to target Epstein-Barr virus (EBV; adjusted $p < 0.0001$) and cytomegalovirus (CMV; adjusted $p = 0.0007$) relative to non-expanded clones (Figure 3G). Many viral infections, including CMV and EBV, may persist as latent infections inside cells after the primary infection has been resolved.[27] To investigate if latent viral infections drove the T cell clonal expansion, we explored the association between TCR diversity and EBV and CMV. As the serostatus for CMV and EBV of the patients was unknown, we constructed a pipeline utilizing Kraken2[28] to detect viral DNA based on WGS data from cell-free DNA in plasma samples. We found DNA evidence of persistent EBV and CMV infections in 43% (51/119) and 54% (64/119) of patients with MIBC, respectively. This matches the reported seroprevalence of CMV (58%)[29] but underestimates that of EBV (95%).[29] Interestingly, patients with detectable CMV DNA had significantly lower TCR diversity ($p = 5.3 \times 10^{-5}$; Figure 3H), while detectable EBV DNA showed no association (Figure S4F). Patients with detectable CMV infection had a higher fraction of their TCRs targeting CMV antigens than those with no detectable CMV (Figure S4G). Latent CMV infection was neither associated with T cell fraction nor disease outcome (Figures 3I, S4H, and S4I). In a linear regression model predicting TCR diversity, both CMV and metastatic disease were significant variables, suggesting that both factors impact TCR diversity independently (Figure 3J). These results indicate that although CMV infection strongly influences diversity, it does not significantly affect MIBC prognosis.

Hyper-expanded clones are highly persistent throughout the disease course

To explore the TCR repertoire during the disease course, we analyzed longitudinal blood samples from a subset of 33 patients with MIBC. Additional TCR-seq was performed on samples taken after chemotherapy, 3 weeks after cystectomy, and either at metastatic relapse or 1 year after cystectomy (3–4 samples per patient). Clones were defined as persistent if found in all available samples, recurrent if found in more than one sample, and transient if found in only one sample. We observed considerable variation across patients at baseline (Figure 4A) and a sharp increase in the prevalence of persistent clones with increasing clone size (Figure S5A). The majority of hyper-expanded clones were expectedly categorized as persistent, while non-expanded clones were commonly transient (Figure 4B). Thus, within the study time frame (median 16 months, range 4–64), almost all hyper-expanded T cell clones detected at baseline remained detectable in circulation. This suggests that the overall T cell landscape remains relatively stable, and significant contractions of hyper-expanded T cell clones are infrequent or occur slowly. However, this observation may partly be influenced by the

CellPress
OPEN ACCESS

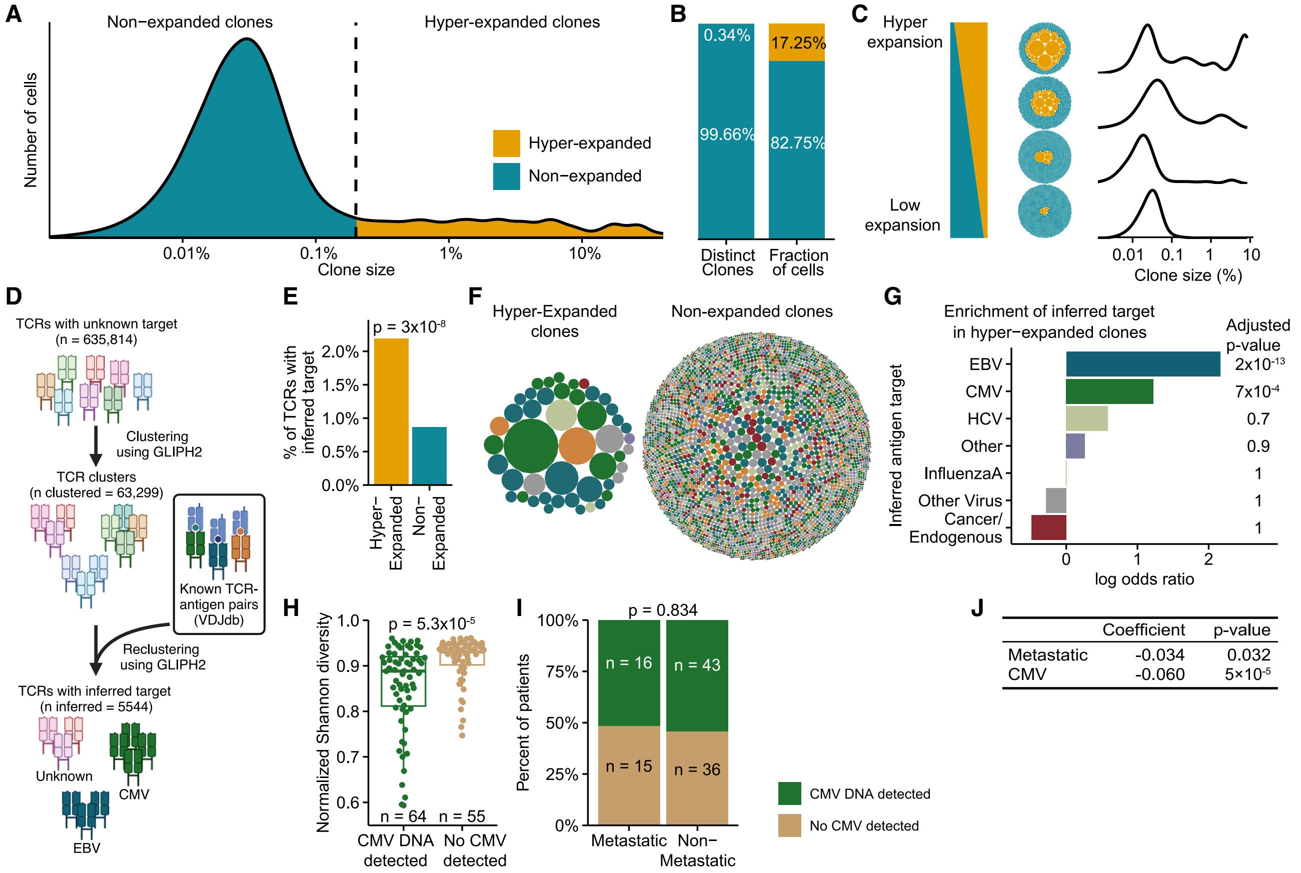

Figure 3. Investigation of clonal expansion in MIBC

(A) Clone size distribution in blood at baseline for patients with MIBC (samples, n = 119; clones, n = 635,814). The density plot is weighted by clone size to represent the distribution of cells.

(B) The clone composition across repertoires as a fraction of unique clones (left) and total relative size of clones (representing the percentage of cells; right).

(C) Visualization of varying levels of clonal expansion using bubble plots and density plots of representative repertoires (patients 82, 8, 102, and 11). Each repertoire is presented as a collection of bubbles with the size of each bubble representing clone size and density plots as described in (A).

(D) Overview of the annotation pipeline of TCR target inference. First, TCR clones were clustered using GLIPH2. Then, TCR clones were reclustered with TCR sequences with known antigen targets from VDJdb using GLIPH2. Last, antigen targets for clusters reemerging in step 2 were inferred (TCR clones in clusters encompassing a TCR with a known antigen were assumed to target the same antigen). n refers to TCR sequences. Created with BioRender.com.

(E) Percentage of TCR clones with inferred antigen target, split by expansion. Test for association between having an inferred target and a clone being expanded (non-expanded, n = 633,663; hyper-expanded, n = 2,151).

(F) Visualization of all TCR clones with inferred targets for hyper-expanded and non-expanded clones.

(G) Test for target enrichment in hyper-expanded clones, based on comparing the number of clones with a given target relative to the total amount of clones, split by expansion (non-expanded, n = 633,663; hyper-expanded, n = 2,151).

(H) Test of difference between normalized Shannon diversity and CMV DNA detection.

(I) Test of difference between the development of metastatic disease and CMV DNA detection.

(J) Coefficients and p values for a linear regression model to predict normalized Shannon diversity based on metastatic disease and CMV detection (MIBC, n = 119). HCV, hepatitis C virus.

See also Figures S3 and S4.

technical explanation that larger clones are more likely to be detected in multiple samples due to their higher frequency. The amount of persistent and recurrent clones was clinically relevant, as the patients who developed metastatic disease had a higher fraction of these clones (Figure 4C). This reflects our observation that low TCR diversity is associated with the development of metastases (Figure S1A), given that persistent and recurrent clones were mostly hyper-expanded, and these correlated negatively with TCR diversity (Figure S4A). Consistent with this, we found that patients with high amounts of persistent and recurrent clones had shorter OS (Figure S5B).

Treatment decreases TCR diversity and lymphocyte counts in patients with good outcomes

We analyzed the dynamic changes to the TCR landscape during and after treatment in patients with metastatic disease and those without, separately. While patients in the non-metastatic group initially exhibited higher TCR diversity, these patients

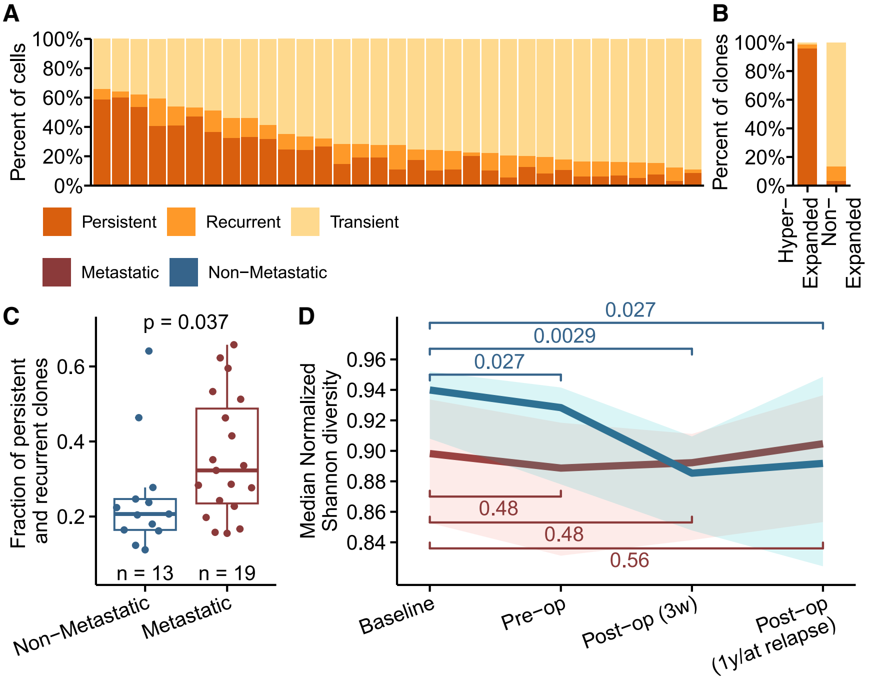

Figure 4. Longitudinal analysis of the TCR landscape in MIBC

(A) Visualization of the abundance of persistent, recurrent, and transient clones at baseline. A bar for each patient (n = 33).

(B) The percentage of hyper-expanded and non-expanded clones divided into persistent, recurrent, and transient clones.

(C) Test of difference between the development of metastatic disease and total relative size of persistent and recurrent clones at baseline (one patient excluded from analysis due to incomplete follow-up).

(D) Lineplot showing the change in median normalized Shannon diversity through treatment for patients with and without metastatic disease. The shadow shows the interquartile range. p values are calculated using paired Wilcoxon signed-rank tests using the individual diversity values at given time points (non-metastatic, n = 13; metastatic, n = 19).

See also Figure S5.

experienced a decrease in diversity during treatment. This was not observed in the group of patients with metastatic disease, resulting in equivalent TCR diversity in both patient groups after cystectomy (Figures 4D and S5C).

To further investigate the dynamics of the immune landscape, we analyzed longitudinal biochemical laboratory measures of blood cell counts from a subset of 58 patients with MIBC. We found that lymphocyte counts, which are primarily T cells, consistently decreased from baseline throughout treatment in patients without metastatic disease, a trend not evident in patients with metastatic disease (Figure S5D). In contrast, neutrophil counts and overall leukocyte counts only decreased after chemotherapy initiation, whereafter they recovered to baseline levels in both patient groups (Figures S5E and S5F). These results indicate that treatment may negatively impact TCR diversity and lymphocyte counts, particularly in good-prognosis patients with high-diversity repertoires.

TCR diversity is associated with outcomes in early-stage bladder cancer

To investigate the impact of TCR diversity on early-stage bladder cancer, we performed TCR-seq on blood samples taken before and after BCG immunotherapy from 30 patients with NMIBC (n, before BCG = 28; n, after BCG = 28; 26 paired samples; Figure 1). Noticeably, TCR diversity was equivalent to that of patients with MIBC (Figure 5A). Outcomes after treatment were dichotomized into either late or no high-grade recurrence (>2 years), or early high-grade recurrence (<2 years) or progression. We observed no significant difference in TCR diversity between the two groups, before or after BCG (Figure 5B). Although we did not find an overall change in diversity after BCG (Figure 5C), we found that TCR diversity increased significantly in patients with late or no high-grade recurrence, contrasting with the chemotherapy-treated MIBC cohort where a decrease in diversity was observed (Figure 5D). Progression-free survival (PFS) was not associated with TCR diversity before BCG (median split; Figure 5E). However, patients with low diversity after BCG had shorter PFS (HR = infinity, p = 0.015; Figure 5F). We estimated the T cell fraction for 110 patients with NMIBC from an extended cohort utilizing WES data applicable for TcellExTRECT and found that patients with a lower T cell fraction had shorter PFS (HR = 3.8, p = 0.027; Figure 5G), which is comparable to the MIBC cohort. As only nine of these patients had TCR-seq data available, we did not attempt a joint analysis of TCR diversity and T cell fraction. However, these findings collectively support that low TCR diversity and a low T cell fraction indicate a poor prognosis in NMIBC.

Peripheral TCR diversity is associated with tumor biology

To evaluate if peripheral TCR diversity was associated with specific tumor biology characteristics, we investigated RNA-seq and WES data from tumor biopsies from patients with MIBC. We performed differential gene expression analysis on the 2,000 most variable genes. Of these, we found that 153 and 39 genes were significantly upregulated in patients with low (n = 23) and high (n = 21) TCR diversity, respectively (Figure S6A). Genes upregulated in patients with low TCR diversity were mainly associated with extracellular matrix organization or signal transduction. Genes upregulated in patients with high TCR diversity were related to the metabolism of proteins or RNA (Figures S6B and S6C). When investigating tumor genetics, we found no association between peripheral TCR diversity and the frequency of common bladder cancer driver genes or the overall tumor mutation burden (Figures S6D and S6E). Lastly, we explored if TCR diversity might affect the ability to detect ctDNA in baseline blood samples. Pre-treatment ctDNA has previously been associated with aggressive disease and increased

CellPress
OPEN ACCESS

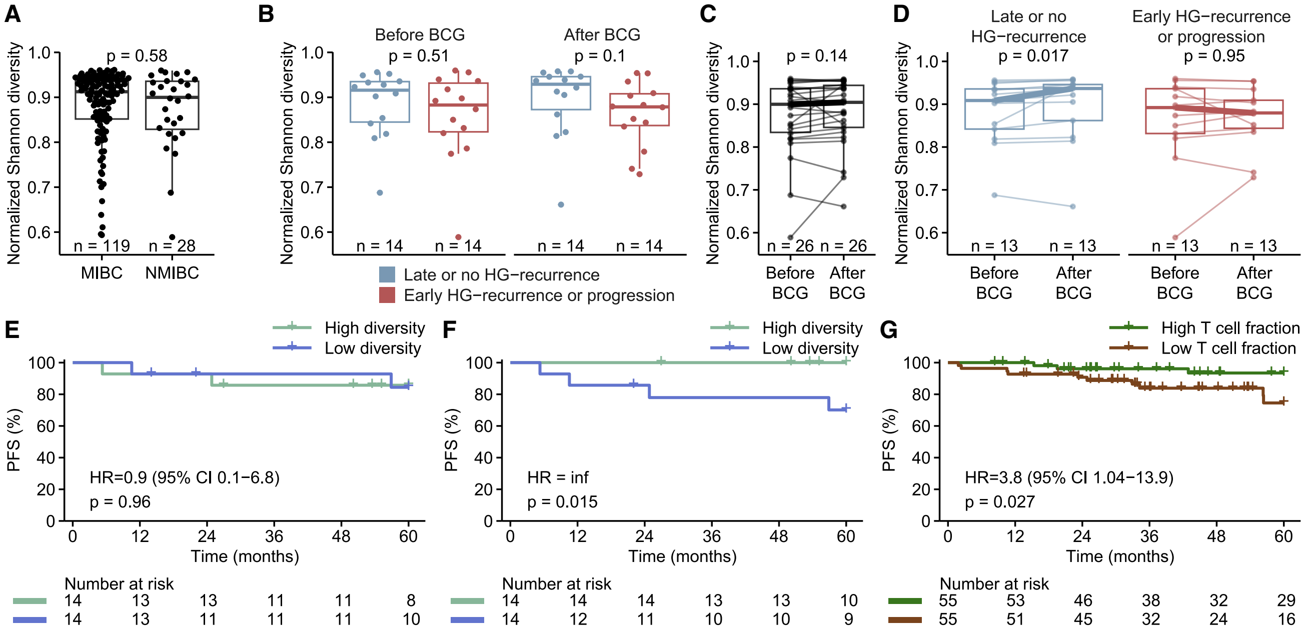

Figure 5. TCR diversity is associated with outcomes in early-stage bladder cancer
(A) Test of difference between baseline normalized Shannon diversity and type of bladder cancer.
(B) Test of difference between normalized Shannon diversity before and after BCG in patients with late or no HG recurrence versus early HG recurrence or progression.
(C) Test of difference in the change in normalized Shannon diversity for each patient before and after BCG.
(D) Test of difference in the change in normalized Shannon diversity for each patient before and after BCG, split by patients with late or no HG recurrence and patients with early HG recurrence or progression.
(E and F) Survival analyses investigating the association of PFS and TCR diversity (median split) before (E) and after (F) BCG.
(G) Survival analysis investigating the association of PFS and relative T cell fraction (median split). T cell fraction was estimated on WES data from an extended cohort of patients with NMIBC (*n* = 110). HG, high grade; CI, confidence interval.

risk of metastatic progression.[16] Interestingly, we found that TCR diversity was significantly lower in the ctDNA-positive group, supporting that patients with low TCR diversity may harbor tumors with an increased risk of metastatic dissemination (Figures S6F).

Tumor TCR repertoires are distinct from peripheral repertoires

The relationship between peripheral blood and tumor TCR repertoires was investigated by performing additional TCR-seq on tumor DNA from 47 patients (MIBC = 28, NMIBC = 19). Tumor TCR repertoires generally had fewer clones and were less diverse than the peripheral blood samples (Figures S6G and S6H). Tumor TCR diversity was not associated with outcome in either cohort (Figures S6I and S6J). Neither the number of clones nor TCR diversity was correlated between tumor and peripheral blood TCR repertoires, indicating that the two repertoires represent different biologies (Figures S6K and S6L). To explore this further, we analyzed the clones that were common between the baseline blood repertoires and the tumor repertoires (Figure 6A). Visualization of the common clones revealed that tumor and blood shared limited amounts of T cell clones (Figure 6B). However, we noticed that larger clones in the blood had an increased tendency to be common, while this was less pronounced for larger clones in the tumor (Figure 6C). To investigate if T cell clones hyper-expanded in the blood were actively migrating to and accumulating in the tumor microenvironment, we compared the sizes of the T cell clones common between blood and tumor. The size of the common clones exhibited a weak correlation between tumor and blood (Figure 6D) and a strong correlation between consecutive blood samples (Figure 6E). Additionally, we quantified significant alterations in clone sizes and found that common clones were generally smaller than expected in the tumor relative to the blood, indicating that hyper-expanded clones infiltrate the tumor less than anticipated at random (Figures 6D and 6E). Together, this indicates that while clones hyper-expanded in the blood are often found in the tumor, they do not seem to be enriched in the tumor microenvironment. Instead, the tumor microenvironment is likely composed mainly of T cells with limited recirculation to the bloodstream.

Single-cell sequencing reveals the T cell subtypes of hyper-expanded and non-expanded clones

To gain information on the T cell subtypes in patients with bladder cancer, we performed single-cell RNA-seq on paired tumor and blood samples. Given the requirement for viable cells, we collected fresh blood and tumor samples from four patients with bladder cancer undergoing cystectomy. Paired TCR and full-length RNA profiling were performed on isolated T cells

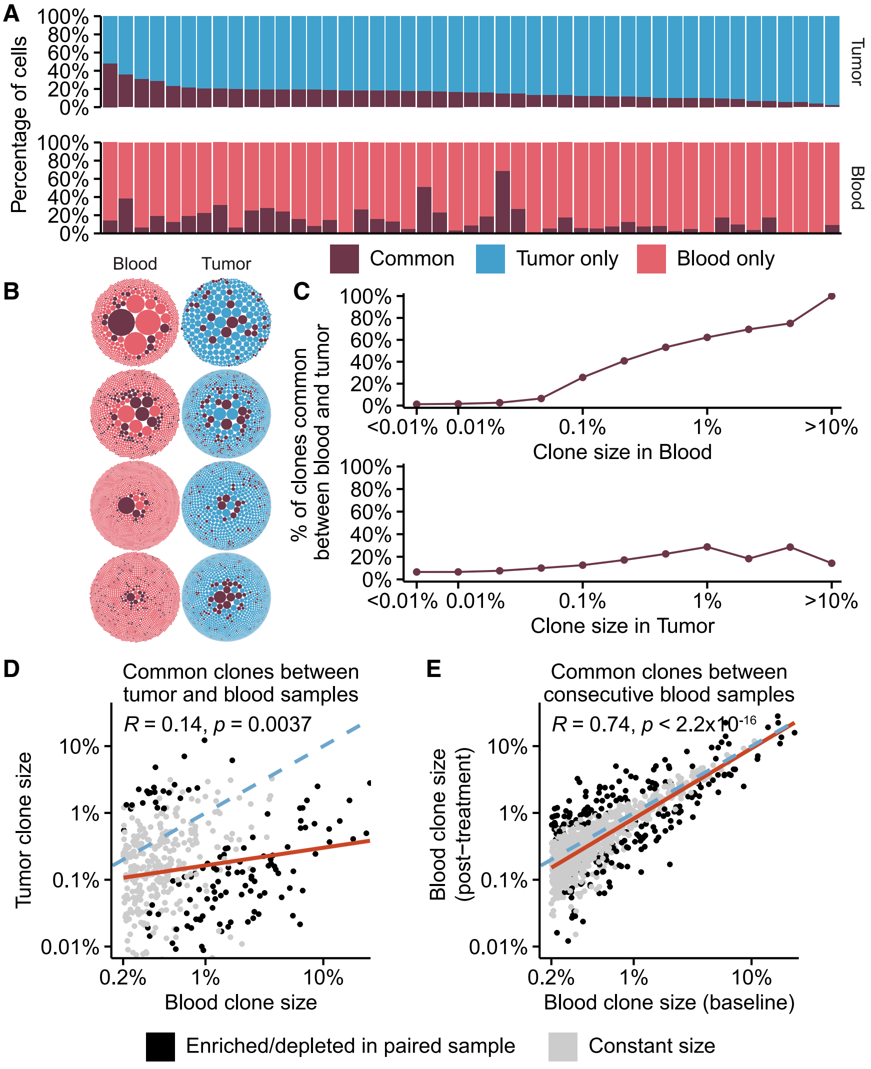

Figure 6. Exploration of tumor TCR repertoires

(A) The degree of overlap between blood and tumor TCR repertoires (representing the relative size of common clones). Vertically aligned bars represent one patient, showing MIBC (n = 28) and NMIBC (n = 19).

(B) Visualization of clones common between blood and tumor repertoires. Horizontally aligned bubble plots represent the same patient (82, 8, 102, and 11).

(C) Distribution of the percentage of clones common between tumor and blood across varying clone sizes in either blood (top) or tumor (bottom; n, common clones = 5,672). Each point represents a uniformly distributed bin (log scale), and the x axis placement indicates the end of the bin.

(D and E) Correlation of size between hyper-expanded clones at baseline shared with either tumor (D, n = 423) or post-treatment blood (E, n = 973; includes after BCG for NMIBC and 3 weeks after cystectomy for MIBC). The blue line indicates a one-to-one linear relationship. The orange line indicates a linear model fit. Dots are colored based on significant enrichment/depletion under the assumption of random clone sharing. The analysis only includes persistent clones.

See also Figure S6.

resulting in cell recovery ranging between 271 and 3,199 cells and clone count varying between 226 and 2,248 clones (Figure S7A). When analyzing T cell clones common between blood and tumor samples, we again found that larger clones in the blood had an increased tendency to be common, whereas this trend was less evident for larger clones in the tumor. This suggests that the likelihood of a clone being common is primarily influenced by its size in peripheral blood (Figures 7A and 7B).

The T cell clones were annotated using CellTypist[30] to identify T cell subtypes revealing a clear contrast between the blood and tumor samples. Regulatory T cells were dominant in the tumor samples, while a combination of naive and cytotoxic T cells was dominant in the blood samples (Figure 7C). More explicitly, the majority of the hyper-expanded clones found in the blood samples were annotated as terminally differentiated effector memory T cells with high levels of cytotoxic genes, whereas the non-expanded clones were largely naive T cells (Figures 7D and S7B). Cytotoxic T cells were most likely to be common between two samples, in both tumor and blood (Figure S7C). A uniform manifold approximation and projection (UMAP) analysis based on the expression data revealed that the T cells clustered primarily based on their subtypes, as annotated by CellTypist (Figures 7E and 7F). The expression of marker genes fits with the T cell subtype annotation (Figure S7D).

To examine if the hyper-expanded T cell clones found by bulk TCR-seq analysis also matched a profile of cytotoxic T cells, we performed RNA-seq on buffy coat samples from eight patients with MIBC, four with high and four with low TCR diversity as measured by bulk TCR-seq. We estimated the immune cell composition of each patient through gene set variation analysis utilizing signatures from Travaglini et al.[31] This revealed a lower naive to effector memory T cell ratio in patients with low TCR diversity relative to patients with high TCR diversity (Figure 7G). This supports that the majority of hyper-expanded clones found in patients with low TCR diversity may primarily be composed of effector memory T cells, as observed in the single-cell data.

DISCUSSION

In this study, we present a comprehensive analysis of the TCR repertoire in patients with bladder cancer. We have characterized the peripheral blood and tumor TCR landscapes and explored the dynamics of peripheral blood TCR repertoires during treatment. Our analysis revealed that both the number of T cells and the diversity of TCR landscapes in peripheral blood are important for the survival of patients with bladder cancer.

CellPress OPEN ACCESS

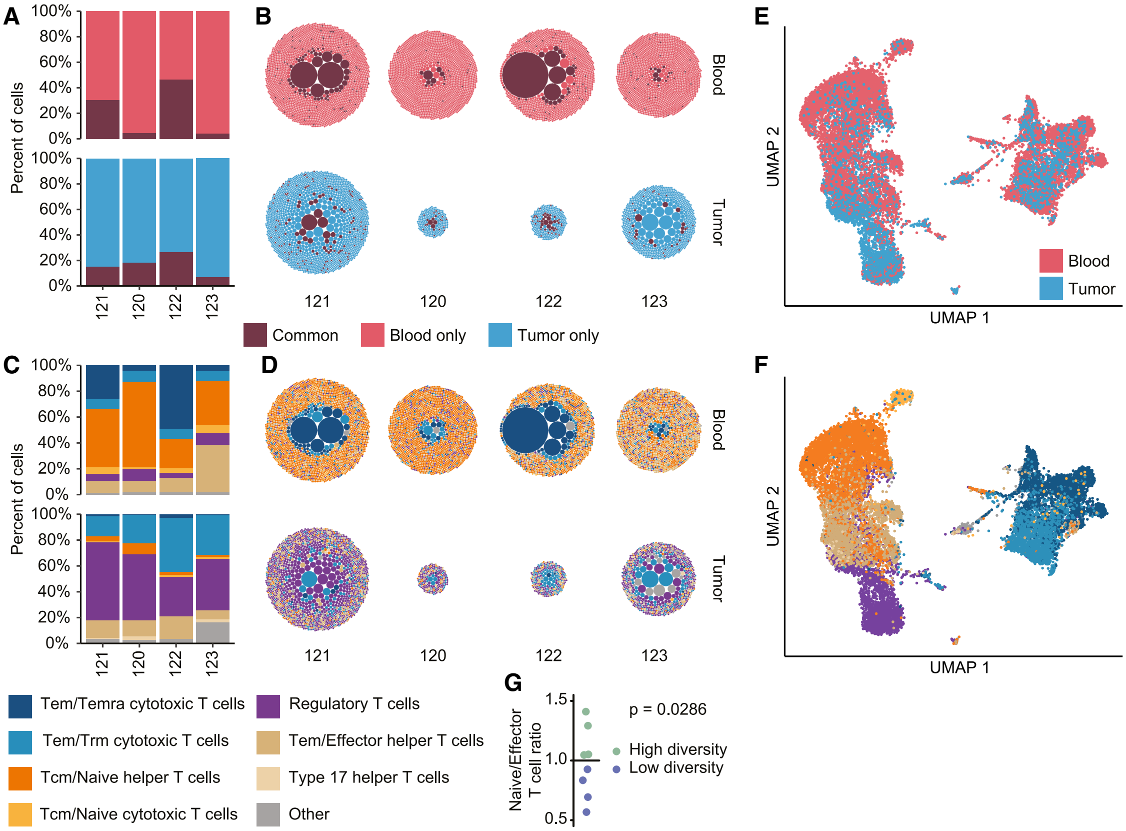

Figure 7. Single-cell sequencing reveals the T cell subtypes of hyper-expanded and non-expanded clones

(A) Relative size of clones common between tumor and blood, and clones unique to tumor or blood for four patients with bladder cancer based on single-cell data.
(B) Visualization of the common clones.
(C) Relative size of eight different T cell subtypes in each sample.
(D) Visualization of the T cell subtypes.
(E and F) UMAP colored by sample type (E) or T cell subtypes (F).
(G) The ratio of naive/effector T cells for eight patients with RNA-seq from blood showing higher ratios in patients with high TCR diversity (n = 4) relative to patients with low TCR diversity (n = 4). Tem, effector memory T cell; Temra, effector memory T cell re-expressing CD45RA; Trm, tissue-resident memory T cell; Tcm, central memory T cell.
See also Figure S7.

We have specifically shown that low TCR diversity and low relative T cell fraction are both associated with poor patient outcomes in MIBC and NMIBC. These findings were validated in independent cohorts from TCGA and IMvigor210. Furthermore, we show that patients with MIBC who later developed metastatic disease had lower TCR diversity at baseline. Interestingly, while chemotherapy efficacy has a well-established association with patient outcome,[32,33] it was not associated with TCR diversity or T cell fraction in our study. These measures likely reflect the immune system's intrinsic ability to constrict cancer growth by targeting cancer neoantigens, which may function independently of the cytotoxic effects of chemotherapy. Higher TCR diversity and T cell fraction presumably increase the likelihood that a given neoantigen will be targeted by one or more matching TCRs. Having multiple TCRs targeting the same neoantigen has previously been associated with improved antitumoral response to immunotherapy.[34] Greater antitumoral T cell activity could improve outcomes by restricting primary tumor growth and limiting metastatic dissemination. Together, our results indicate that TCR diversity is likely a generalizable measure of the state of the immune system, which may impact cancer outcomes.

In our study, hyper-expanded T cell clones were inferred to disproportionately target EBV and CMV antigens. CMV has previously been reported to promote the deterioration of T cell immunity by the formation of hyper-expanded T cell clones,[35] and we showed that the detection of CMV DNA was associated with decreased diversity. Although CMV strongly reduced diversity, CMV DNA detection was not associated with outcome. Previous studies have shown that CMV infection leads to hyper-expansion of clones, but it does not detrimentally affect the remaining T cell structure or impair the acute immune response to other pathogens.[36,37] However, CMV has previously been linked to increased mortality,[38] indicating some immune-impairing effects. The absence of a discernible effect of EBV on TCR diversity could be caused

by reduced sensitivity to detect EBV infections. Although EBV has been associated with cancer formation,[39] most individuals have previously been infected with EBV,[29] making quantification of its effect challenging in the present study. Together, this supports that further studies of viral infections and their impact on the immune system and on oncological treatment regimens are urgently needed.

Based on longitudinal analysis, we observed that patients with MIBC who did not develop metastases showed a significant decline in peripheral blood TCR diversity and lymphocyte counts. These measures did not fully recover during the study, indicating that these patients may have suffered permanent treatment-induced immune degradation. In contrast, no systematic changes were observed in patients developing metastatic disease. This suggests that while patients with higher TCR diversity may be less likely to develop metastases, their TCR landscapes are more affected by treatment. Cisplatin-based chemotherapy, while highly effective against cancer cells, is known to induce lymphopenia through cytotoxic effects on T cells.[40] This likely causes a decrease in TCR diversity through depletion of non-expanded clones, while expanded clones may be reduced in absolute numbers of circulating T cells but are unlikely to be eliminated. Our single-cell analysis showed that non-expanded clones were primarily naive T cells, and the loss of these cells reduces the immune system's ability to generate responses to new pathogens. This could lead to an increased risk of severe infections and risk of developing other cancers.[41] Our TCR data are limited to 1 year after cystectomy, but if these effects on the TCR landscape are permanent, they could cause detrimental effects on long-term patient health.[42] We did not observe such a decrease in TCR diversity for patients who developed metastases. These patients tend to have more hyper-expanded clones, which are unlikely to be completely eradicated by chemotherapy, hereby causing the decrease in TCR diversity through treatment to be minimal. Conversely, for patients with early-stage bladder cancer, we found that patients with good outcomes after BCG showed an increase in TCR diversity. Similar effects were observed in patients with glioblastoma receiving oncolytic therapy in a previous study.[43] This increase may stem from the non-tumor-specific immune stimulation by BCG, reflecting an important functional aspect of BCG response. We only found an association between TCR diversity and PFS after BCG treatment. This is supported by previous work, where tumor and urine samples taken after BCG treatment showed differences between BCG responders and non-responders.[1] Taken together, this work suggests that a level of restraint should be considered when administering chemotherapy. Particularly, future clinical trials should investigate if increased surveillance, e.g., using ctDNA to identify early relapse,[16] might be a suitable alternative for patients with an otherwise good prognosis and high TCR diversity. While we find an association between low TCR diversity and poor outcomes in bladder cancer, our study has limited power to determine the cause/effect relationship between TCR diversity and disease progression. However, we observed no difference in TCR diversity between different T and N stages in MIBC, nor between MIBC and NMIBC. Together with the lack of systematic changes in TCR diversity over time in patients developing metastatic disease, this indicates that disease progression has little impact on TCR diversity.

TCR diversity in circulation also affected tumor biology. We showed that genes with higher expression in patients with low TCR diversity were mainly related to extracellular matrix organization. Remodeling of the extracellular matrix is known to be related to cancer progression and metastatic disease,[44] which supports our finding that low TCR diversity is associated with poor outcomes. In addition, we showed that the TCR repertoire found in circulation was distinct from the repertoire found within the tumor. Using single-cell analysis, we showed that clones common between tumor and blood were mainly cytotoxic T cells, consistent with previous work in patients with renal and lung cancers.[45] Furthermore, we demonstrated that the hyper-expanded clones found in circulation were dominated by cytotoxic T cells, which mostly were in a terminally differentiated state. Conversely, non-expanded clones were mainly naive T cells, which could indicate higher thymic activity with ongoing production of new T cells in patients with high TCR diversity.

Collectively, we provide evidence that the T cell repertoire in circulation is highly diverse among patients and is associated with outcome—indicating a potential impact on cancer development. Considering the general health relevance of a well-functioning immune system, these findings may have significant clinical implications on risk stratification and treatment sequencing. Immune-competent patients with high TCR diversity might benefit from immune-boosting therapies such as immune checkpoint inhibitors, improving their outcomes even further. In addition, chemotherapy strategies should potentially be used primarily in patients with low TCR diversity and hence low immune competence. Indeed, TCR diversity and other methods to assess patient immune competency may be highly predictive of immunotherapy response, given that a competent immune system is likely a prerequisite for mounting an anti-cancer response. Thus, developing accurate measures to assess immune competency may significantly improve patient stratification for anti-cancer therapies across various cancer types. Future clinical trials should focus on integrating immune competency measures into precision medicine approaches to optimize anti-cancer therapies and patient survival.

Limitations of the study

For the validation analyses in TCGA and IMvigor210, TCR diversity was estimated using germline WES data as TCR-seq data were not available, resulting in lower recovery of T cell clones. TCR target inference was done using CDR3β, which is less accurate compared to using both chains. Although CDR3β is considered to be the major contact point between TCR and antigen, the specificity is influenced by the paired α chain as well as the full β chain. Additionally, the ability to infer targets is limited by the availability of TCR-antigen pairs, most of which are viral. Only a subset of TCRs can be annotated as the majority of the TCR-antigen space is still unknown. While EBV has been associated with cancer formation,[39] the absence of a discernible effect in the present study may be due to its endemic status in the population,[29] making quantification of its effect challenging.

RESOURCE AVAILABILITY

Lead contact

Further information and requests for resources and reagents should be directed to and will be fulfilled by the lead contact, Professor Lars Dyrskjøt (lars@clin.au.dk).

Materials availability

No new reagents were made in this study.

Data and code availability

- Raw sequencing data are deposited at the European Genome-Phenome Archive (EGA), which is hosted by the European Bioinformatics Institute and the Center for Genomic Regulation. The data are available under controlled access at EGA due to privacy laws and legal restrictions associated with sharing sensitive data under the General Data Protection Regulation. Access to data that are under controlled access requires that the data requestor (the legal entity) enter into collaboration and data processing agreements with the Central Denmark Region (the legal entity controlling and being responsible for the data). Request to access data furthermore requires that the purpose of data re-analysis is approved by the Danish National Committee on Health Research Ethics. Upon request, the authors, on behalf of the Central Denmark Region, will enter into a collaboration with the data requestor to apply for approval. Any requests will be reviewed within a time frame of 2–3 weeks by the data assessment committee. This applies to the following datasets: TCR-seq data are available under accession number EGAS50000000940. RNA-seq data from blood samples are available under accession number EGAS50000000939. Single-cell sequencing data, including RNA-seq and TCR-seq data, are available under accession number EGAS50000000938. Clinical information and laboratory blood measurements are provided in Table S3. Processed data produced for this publication, including TCR-seq, RNA-seq, and single-cell RNA-seq, and summary data to create all figures are available as Datas S1 and S2 and Table S3.
- All original code has been deposited at Zenodo and is publicly available at https://doi.org/10.5281/zenodo.14823577 as of the date of publication.
- Any additional information required to reanalyze the data reported in this paper is available from the lead contact upon request.

ACKNOWLEDGMENTS

All of the computing for this project was performed on the GenomeDK cluster. We thank GenomeDK and Aarhus University for providing computational resources and support that contributed to these research results. The authors acknowledge funding received in support of the project from AstraZeneca, the Danish Cancer Society, the Novo Nordisk Foundation, the Lundbeck Foundation, and the Aarhus University Research Foundation.

AUTHOR CONTRIBUTIONS

A.K., N.K., and R.I.J. contributed equally. N.J.B. and L.D. conceived the study design. A.K., N.K., R.I.J., I.N., and D.R. performed data integration. A.K., N.K., R.I.J., and J.A. performed statistical analyses. I.N. coordinated and supervised bulk TCR-seq. N.K. performed single-cell sequencing and RNA-seq on blood samples. K.B.-D. and T.S. selected samples for bulk TCR-seq and collected clinical follow-up information. J.B.J. provided samples for single-cell sequencing. A.K., N.K., R.I.J., N.J.B., and L.D. drafted the manuscript. N.J.B. and L.D. supervised the study. All authors provided feedback and interpretation of results, and all authors approved the final version of the manuscript.

DECLARATION OF INTERESTS

L.D. has sponsored research agreements with C2i Genomics, Natera, AstraZeneca, Photocure, and Ferring and has an advisory/consulting role at Ferring, MSD, Cystotech, and UroGen. L.D. has received speaker honoraria from AstraZeneca, Pfizer, and Roche and travel support from MSD. L.D. is a board member at BioXpedia.

N.J.B. is listed as a co-inventor on a patent to identify responders to cancer treatment (PCT/GB2018/051912) and has a patent application (PCT/GB2020/050221) on methods for cancer prognostication and a patent on methods for predicting anti-cancer response (US14/466,208).

J.B.J. is a member of Advisory Boards at Ferring, Roche, Cepheid, Urotech, Olympus, AMBU, Janssen, and Cystotech, is a speaker at medac, Olympus, Intuitive Surgery, and Photocure ASA, and has research collaborations with medac, Photocure ASA, Roche, Ferring, Olympus, Intuitive Surgery, Astellas, Cepheid, Nucleix, Urotech, Pfizer, AstraZeneca, MeqNordic, Laborie, VingMed, AMBU, and Cystotech.

H.J.W.L.A. has received personal fees and stock from Onc.AI, Sphera, and Love Health and speaking honoraria from Bristol Myers Squibb.

C.A. reports employment at AstraZeneca and has shares in AstraZeneca. C.A. is an inventor of a European patent application relating to assay technology to detect tumor recurrence (PCT/GB2017/053289). This patent has been licensed to commercial entities and, under their terms of employment, C.A. is due a share of any revenue from such license(s). C.A. declares a patent application (PCT/US2017/028013) for methods to detect lung cancer. C.A. is named inventor on a patent application to determine methods and systems for tumor monitoring (PCT/EP2022/077987). C.A. is named inventor on provisional patent protection related to a ctDNA detection algorithm.

D.H. reports employment at AstraZeneca and has shares in AstraZeneca.

STAR★METHODS

Detailed methods are provided in the online version of this paper and include the following:

- KEY RESOURCES TABLE
- EXPERIMENTAL MODEL AND STUDY PARTICIPANT DETAILS
  - Human participants
- METHOD DETAILS
  - DNA and RNA extraction
  - Library preparation and sequencing
  - TCR-seq data analyses
  - RNA-seq data analyses
  - WES data analyses
  - WGS data analyses
  - Annotation of TCR sequences
  - Validation datasets
  - Single-cell analyses
- QUANTIFICATION AND STATISTICAL ANALYSIS

SUPPLEMENTAL INFORMATION

Supplemental information can be found online at https://doi.org/10.1016/j.xcrm.2025.102101.

Received: August 30, 2024
Revised: November 20, 2024
Accepted: April 9, 2025
Published: May 1, 2025

REFERENCES

1. Strandgaard, T., Lindskrog, S.V., Nordentoft, I., Christensen, E., Birkenkamp-Demtröder, K., Andreasen, T.G., Lamy, P., Kjær, A., Ranti, D., Wang, Y.A., et al. (2022). Elevated T-cell Exhaustion and Urinary Tumor DNA Levels Are Associated with Bacillus Calmette-Guérin Failure in Patients with Non-muscle-invasive Bladder Cancer. Eur. Urol. *82*, 646–656.
2. Sokač, M., Ahrenfeldt, J., Litchfield, K., Watkins, T.B.K., Knudsen, M., Dyrskjøt, L., Jakobsen, M.R., and Birkbak, N.J. (2022). Classifying cGAS-STING Activity Links Chromosomal Instability with Immunotherapy

Response in Metastatic Bladder Cancer. Cancer Res. Commun. 2, 762–771.

3. Schumacher, T.N., and Thommen, D.S. (2022). Tertiary lymphoid structures in cancer. Science 375, eabf9419.
4. Chalmers, Z.R., Connelly, C.F., Fabrizio, D., Gay, L., Ali, S.M., Ennis, R., Schrock, A., Campbell, B., Shlien, A., Chmielecki, J., et al. (2017). Analysis of 100,000 human cancer genomes reveals the landscape of tumor mutational burden. Genome Med. 9, 34.
5. Cambier, S., Sylvester, R.J., Collette, L., Gontero, P., Brausi, M.A., van Andel, G., Kirkels, W.J., Silva, F.C.D., Oosterlinck, W., Prescott, S., et al. (2016). EORTC Nomograms and Risk Groups for Predicting Recurrence, Progression, and Disease-specific and Overall Survival in Non-Muscle-invasive Stage Ta-T1 Urothelial Bladder Cancer Patients Treated with 1-3 Years of Maintenance Bacillus Calmette-Guérin. Eur. Urol. 69, 60–69.
6. Witjes, J.A., Bruins, H.M., Cathomas, R., Compérat, E.M., Cowan, N.C., Gakis, G., Hernández, V., Linares Espinós, E., Lorch, A., Neuzillet, Y., et al. (2021). European Association of Urology Guidelines on Muscle-invasive and Metastatic Bladder Cancer: Summary of the 2020 Guidelines. Eur. Urol. 79, 82–104.
7. Alfred Witjes, J., Max Bruins, H., Carrión, A., Cathomas, R., Compérat, E., Efstathiou, J.A., Fietkau, R., Gakis, G., Lorch, A., Martini, A., et al. (2023). European Association of Urology Guidelines on Muscle-invasive and Metastatic Bladder Cancer: Summary of the 2023 Guidelines. Eur. Urol. 85, 17–31. https://doi.org/10.1016/j.eururo.2023.08.016.
8. Powles, T., Valderrama, B.P., Gupta, S., Bedke, J., Kikuchi, E., Hoffman-Censits, J., Iyer, G., Vulsteke, C., Park, S.H., Shin, S.J., et al. (2024). Enfortumab Vedotin and Pembrolizumab in Untreated Advanced Urothelial Cancer. N. Engl. J. Med. 390, 875–888.
9. Charles, J., Mouret, S., Challende, I., Leccia, M.-T., De Fraipont, F., Perez, S., Plantier, N., Plumas, J., Manuel, M., Chaperot, L., and Aspord, C. (2020). T-cell receptor diversity as a prognostic biomarker in melanoma patients. Pigment Cell Melanoma Res. 33, 612–624.
10. Guo, L., Bi, X., Li, Y., Wen, L., Zhang, W., Jiang, W., Ma, J., Feng, L., Zhang, K., and Shou, J. (2020). Characteristics, dynamic changes, and prognostic significance of TCR repertoire profiling in patients with renal cell carcinoma. J. Pathol. 251, 26–37.
11. Cui, J.-H., Lin, K.-R., Yuan, S.-H., Jin, Y.-B., Chen, X.-P., Su, X.-K., Jiang, J., Pan, Y.-M., Mao, S.-L., Mao, X.-F., and Luo, W. (2018). TCR Repertoire as a Novel Indicator for Immune Monitoring and Prognosis Assessment of Patients With Cervical Cancer. Front. Immunol. 9, 2729.
12. Liu, Y.-Y., Yang, Q.-F., Yang, J.-S., Cao, R.-B., Liang, J.-Y., Liu, Y.-T., Zeng, Y.-L., Chen, S., Xia, X.-F., Zhang, K., and Liu, L. (2019). Characteristics and prognostic significance of profiling the peripheral blood T-cell receptor repertoire in patients with advanced lung cancer. Int. J. Cancer 145, 1423–1431.
13. Han, J., Duan, J., Bai, H., Wang, Y., Wan, R., Wang, X., Chen, S., Tian, Y., Wang, D., Fei, K., et al. (2020). TCR Repertoire Diversity of Peripheral PD-1CD8 T Cells Predicts Clinical Outcomes after Immunotherapy in Patients with Non-Small Cell Lung Cancer. Cancer Immunol. Res. 8, 146–154.
14. Manuel, M., Tredan, O., Bachelot, T., Clapisson, G., Courtier, A., Parmentier, G., Rabeony, T., Grives, A., Perez, S., Mouret, J.-F., et al. (2012). Lymphopenia combined with low TCR diversity (divpenia) predicts poor overall survival in metastatic breast cancer patients. OncoImmunology 1, 432–440.
15. Nishida, J., Cristea, S., Bodapati, S., Puleo, J., Bai, G., Patel, A., Hughes, M., Snow, C., Borges, V., Ruddy, K.J., et al. (2023). Peripheral blood TCR clonotype diversity as an age-associated marker of breast cancer progression. Proc. Natl. Acad. Sci. USA 120, e2316763120.
16. Christensen, E., Birkenkamp-Demtröder, K., Sethi, H., Shchegrova, S., Salari, R., Nordentoft, I., Wu, H.-T., Knudsen, M., Lamy, P., Lindskrog, S.V., et al. (2019). Early Detection of Metastatic Relapse and Monitoring of Therapeutic Efficacy by Ultra-Deep Sequencing of Plasma Cell-Free DNA in Patients With Urothelial Bladder Carcinoma. J. Clin. Oncol. 37, 1547–1557.
17. Christensen, E., Nordentoft, I., Birkenkamp-Demtroder, K., Elbæk, S.K., Lindskrog, S.V., Taber, A., Andreasen, T.G., Strandgaard, T., Knudsen, M., Lamy, P., et al. (2023). Cell-free urine- and plasma DNA mutational analysis predicts neoadjuvant chemotherapy response and outcome in patients with muscle invasive bladder cancer. Clin. Cancer Res. 29, 1582–1591. https://doi.org/10.1158/1078-0432.CCR-22-3250.
18. Lindskrog, S.V., Birkenkamp-Demtroder, K., Nordentoft, I., Laliotis, G., Lamy, P., Christensen, E., Renner, D., Andreasen, T.G., Lange, N., Sharma, S., et al. (2023). Circulating tumor DNA analysis in advanced urothelial carcinoma: insights from biological analysis and extended clinical follow-up. Clin. Cancer Res. 29, 4797–4807. https://doi.org/10.1158/1078-0432.CCR-23-1860.
19. Nordentoft, I., Lindskrog, S.V., Birkenkamp-Demtröder, K., Gonzalez, S., Kuzman, M., Levatic, J., Glavas, D., Ptashkin, R., Smadbeck, J., Afterman, D., et al. (2024). Whole-genome Mutational Analysis for Tumor-informed Detection of Circulating Tumor DNA in Patients with Urothelial Carcinoma. Eur. Urol. 86, 301–311. https://doi.org/10.1016/j.eururo.2024.05.014.
20. Bentham, R., Litchfield, K., Watkins, T.B.K., Lim, E.L., Rosenthal, R., Martínez-Ruiz, C., Hiley, C.T., Bakir, M.A., Salgado, R., Moore, D.A., et al. (2021). Using DNA sequencing data to quantify T cell fraction and therapy response. Nature 597, 555–560.
21. Robertson, A.G., Kim, J., Al-Ahmadie, H., Bellmunt, J., Guo, G., Cherniack, A.D., Hinoue, T., Laird, P.W., Hoadley, K.A., Akbani, R., et al. (2017). Comprehensive Molecular Characterization of Muscle-Invasive Bladder Cancer. Cell 171, 540–556.e25.
22. Mariathasan, S., Turley, S.J., Nickles, D., Castiglioni, A., Yuen, K., Wang, Y., Kadel, E.E., III, Koeppen, H., Astarita, J.L., Cubas, R., et al. (2018). TGFβ attenuates tumour response to PD-L1 blockade by contributing to exclusion of T cells. Nature 554, 544–548.
23. Joshi, K., de Massy, M.R., Ismail, M., Reading, J.L., Uddin, I., Woolston, A., Hatipoglu, E., Oakes, T., Rosenthal, R., Peacock, T., et al. (2019). Spatial heterogeneity of the T cell receptor repertoire reflects the mutational landscape in lung cancer. Nat. Med. 25, 1549–1559.
24. Bick, A.G., Weinstock, J.S., Nandakumar, S.K., Fulco, C.P., Bao, E.L., Zekavat, S.M., Szeto, M.D., Liao, X., Leventhal, M.J., Nasser, J., et al. (2020). Inherited causes of clonal haematopoiesis in 97,691 whole genomes. Nature 586, 763–768.
25. Huang, H., Wang, C., Rubelt, F., Scriba, T.J., and Davis, M.M. (2020). Analyzing the Mycobacterium tuberculosis immune response by T-cell receptor clustering with GLIPH2 and genome-wide antigen screening. Nat. Biotechnol. 38, 1194–1202.
26. Goncharov, M., Bagaev, D., Shcherbinin, D., Zvyagin, I., Bolotin, D., Thomas, P.G., Minervina, A.A., Pogorelyy, M.V., Ladell, K., McLaren, J. E., et al. (2022). VDJdb in the pandemic era: a compendium of T cell receptors specific for SARS-CoV-2. Nat. Methods 19, 1017–1019.
27. Cohen, J.I. (2020). Herpesvirus latency. J. Clin. Investig. 130, 3361–3369.
28. Wood, D.E., Lu, J., and Langmead, B. (2019). Improved metagenomic analysis with Kraken 2. Genome Biol. 20, 257.
29. Mentzer, A.J., Brenner, N., Allen, N., Littlejohns, T.J., Chong, A.Y., Cortes, A., Almond, R., Hill, M., Sheard, S., McVean, G., et al. (2022). Identification of host–pathogen-disease relationships using a scalable multiplex serology platform in UK Biobank. Nat. Commun. 13, 1818–1912.
30. Domínguez Conde, C., Xu, C., Jarvis, L.B., Rainbow, D.B., Wells, S.B., Gomes, T., Howlett, S.K., Suchanek, O., Polanski, K., King, H.W., et al. (2022). Cross-tissue immune cell analysis reveals tissue-specific features in humans. Science 376, eabl5197.
31. Travaglini, K.J., Nabhan, A.N., Penland, L., Sinha, R., Gillich, A., Sit, R.V., Chang, S., Conley, S.D., Mori, Y., Seita, J., et al. (2020). A molecular cell atlas of the human lung from single-cell RNA sequencing. Nature 587, 619–625.
32. Grossman, H.B., Natale, R.B., Tangen, C.M., Speights, V.O., Vogelzang, N.J., Trump, D.L., deVere White, R.W., Sarosdy, M.F., Wood, D.P., Jr., Raghavan, D., and Crawford, E.D. (2003). Neoadjuvant chemotherapy plus

CellPress
OPEN ACCESS

cystectomy compared with cystectomy alone for locally advanced bladder cancer. N. Engl. J. Med. *349*, 859–866.

33. Advanced Bladder Cancer Meta-analysis Collaboration (2004). Neo-adjuvant Chemotherapy for Invasive Bladder Cancer (Cochrane Libr). https://doi.org/10.1002/14651858.cd005246.
34. Puig-Saus, C., Sennino, B., Peng, S., Wang, C.L., Pan, Z., Yuen, B., Purandare, B., An, D., Quach, B.B., Nguyen, D., et al. (2023). Neoantigen-targeted CD8+ T cell responses with PD-1 blockade therapy. Nature *615*, 697–704.
35. Zangger, N., and Oxenius, A. (2022). T cell immunity to cytomegalovirus infection. Curr. Opin. Immunol. *77*, 102185.
36. van den Berg, S.P.H., Lanfermeijer, J., Jacobi, R.H.J., Hendriks, M., Vos, M., van Schuijlenburg, R., Nanlohy, N.M., Borghans, J.A.M., van Beek, J., van Baarle, D., and de Wit, J. (2021). Latent CMV infection is associated with lower influenza virus-specific memory T-cell frequencies, but not with an impaired T-cell response to acute influenza virus infection. Front. Immunol. *12*, 663664.
37. Lindau, P., Mukherjee, R., Gutschow, M.V., Vignali, M., Warren, E.H., Riddell, S.R., Makar, K.W., Turtle, C.J., and Robins, H.S. (2019). Cytomegalovirus exposure in the elderly does not reduce CD8 T cell repertoire diversity. J. Immunol. *202*, 476–483.
38. Gkrania-Klotsas, E., Langenberg, C., Sharp, S.J., Luben, R., Khaw, K.-T., and Wareham, N.J. (2013). Seropositivity and higher immunoglobulin g antibody levels against cytomegalovirus are associated with mortality in the population-based European prospective investigation of Cancer-Norfolk cohort. Clin. Infect. Dis. *56*, 1421–1427.
39. Farrell, P.J. (2019). Epstein-Barr Virus and Cancer. Annu. Rev. Pathol. *14*, 29–53.
40. Prestayko, A.W., D'Aoust, J.C., Issell, B.F., and Crooke, S.T. (1979). Cisplatin (cis-diamminedichloroplatinum II). Cancer Treat Rev. *6*, 17–39.
41. Lian, J., Yue, Y., Yu, W., and Zhang, Y. (2020). Immunosenescence: a key player in cancer development. J. Hematol. Oncol. *13*, 151.
42. Kooshesh, K.A., Foy, B.H., Sykes, D.B., Gustafsson, K., and Scadden, D. T. (2023). Health Consequences of Thymus Removal in Adults. N. Engl. J. Med. *389*, 406–417.
43. Ling, A.L., Solomon, I.H., Landivar, A.M., Nakashima, H., Woods, J.K., Santos, A., Masud, N., Fell, G., Mo, X., Yilmaz, A.S., et al. (2023). Clinical trial links oncolytic immunoactivation to survival in glioblastoma. Nature *623*, 157–166.
44. Winkler, J., Abisoye-Ogunniyan, A., Metcalf, K.J., and Werb, Z. (2020). Concepts of extracellular matrix remodelling in tumour progression and metastasis. Nat. Commun. *11*, 5120.
45. Wu, T.D., Madireddi, S., de Almeida, P.E., Banchereau, R., Chen, Y.-J.J., Chitre, A.S., Chiang, E.Y., Iftikhar, H., O'Gorman, W.E., Au-Yeung, A., et al. (2020). Peripheral T cell expansion predicts tumour infiltration and clinical response. Nature *579*, 274–278.
46. Subramanian, A., Tamayo, P., Mootha, V.K., Mukherjee, S., Ebert, B.L., Gillette, M.A., Paulovich, A., Pomeroy, S.L., Golub, T.R., Lander, E.S., and Mesirov, J.P. (2005). Gene set enrichment analysis: a knowledge-based approach for interpreting genome-wide expression profiles. Proc. Natl. Acad. Sci. USA *102*, 15545–15550.
47. Liberzon, A., Subramanian, A., Pinchback, R., Thorvaldsdóttir, H., Tamayo, P., and Mesirov, J.P. (2011). Molecular signatures database (MSigDB) 3.0. Bioinformatics *27*, 1739–1740.
48. Shugay, M., Bagaev, D.V., Zvyagin, I.V., Vroomans, R.M., Crawford, J.C., Dolton, G., Komech, E.A., Sycheva, A.L., Koneva, A.E., Egorov, E.S., et al. (2018). VDJdb: a curated database of T-cell receptor sequences with known antigen specificity. Nucleic Acids Res. *46*, D419–D427.
49. Bolotin, D.A., Poslavsky, S., Mitrophanov, I., Shugay, M., Mamedov, I.Z., Putintseva, E.V., and Chudakov, D.M. (2015). MiXCR: software for comprehensive adaptive immunity profiling. Nat. Methods *12*, 380–381.
50. Bolotin, D.A., Poslavsky, S., Davydov, A.N., Frenkel, F.E., Fanchi, L., Zolotareva, O.I., Hemmers, S., Putintseva, E.V., Obraztsova, A.S., Shugay, M., et al. (2017). Antigen receptor repertoire profiling from RNA-seq data. Nat. Biotechnol. *35*, 908–911.
51. Patro, R., Duggal, G., Love, M.I., Irizarry, R.A., and Kingsford, C. (2017). Salmon provides fast and bias-aware quantification of transcript expression. Nat. Methods *14*, 417–419.
52. R Core Team (2020). R: A Language and Environment for Statistical Computing (Preprint at R Foundation for Statistical Computing).
53. Love, M.I., Huber, W., and Anders, S. (2014). Moderated estimation of fold change and dispersion for RNA-seq data with DESeq2. Genome Biol. *15*, 550.
54. Rainer, J. (2017). EnsDb.Hsapiens.v86: Ensembl based annotation package. R package version 2.90.0. Bioconductor. https://doi.org/10.18129/B9.BIOC.ENSDB.HSAPIENS.V86.
55. Yu, G., and He, Q.-Y. (2016). ReactomePA: an R/Bioconductor package for reactome pathway analysis and visualization. Mol. Biosyst. *12*, 477–479.
56. Bray, N.L., Pimentel, H., Melsted, P., and Pachter, L. (2016). Near-optimal probabilistic RNA-seq quantification. Nat. Biotechnol. *34*, 525–527.
57. Robinson, M.D., McCarthy, D.J., and Smyth, G.K. (2010). edgeR: a Bioconductor package for differential expression analysis of digital gene expression data. Bioinformatics *26*, 139–140.
58. Hänzelmann, S., Castelo, R., and Guinney, J. (2013). GSVA: gene set variation analysis for microarray and RNA-seq data. BMC Bioinf. *14*, 7.
59. McKenna, A., Hanna, M., Banks, E., Sivachenko, A., Cibulskis, K., Kernytsky, A., Garimella, K., Altshuler, D., Gabriel, S., Daly, M., and DePristo, M. A. (2010). The Genome Analysis Toolkit: a MapReduce framework for analyzing next-generation DNA sequencing data. Genome Res. *20*, 1297–1303.
60. Cibulskis, K., Lawrence, M.S., Carter, S.L., Sivachenko, A., Jaffe, D., Sougnez, C., Gabriel, S., Meyerson, M., Lander, E.S., and Getz, G. (2013). Sensitive detection of somatic point mutations in impure and heterogeneous cancer samples. Nat. Biotechnol. *31*, 213–219.
61. Cingolani, P., Platts, A., Wang, L.L., Coon, M., Nguyen, T., Wang, L., Land, S.J., Lu, X., and Ruden, D.M. (2012). A program for annotating and predicting the effects of single nucleotide polymorphisms, SnpEff: SNPs in the genome of Drosophila melanogaster strain w1118. Fly *6*, 80–92 iso-2; iso-3. .
62. Wang, K., Li, M., and Hakonarson, H. (2010). ANNOVAR: functional annotation of genetic variants from high-throughput sequencing data. Nucleic Acids Res. *38*, e164.
63. Knaus, B.J., and Grünwald, N.J. (2017). vcfr: a package to manipulate and visualize variant call format data in R. Mol. Ecol. Resour. *17*, 44–53.
64. M. Dowle and A. Srinivasan (2023). data.table: Extension of 'data.frame'. R package version 1.14.8, https://CRAN.R-project.org/package=data.table
65. Wickham, H., Averick, M., Bryan, J., Chang, W., McGowan, L., François, R., Grolemund, G., Hayes, A., Henry, L., Hester, J., et al. (2019). Welcome to the tidyverse. J. Open Source Softw. *4*, 1686.
66. Kim, S., Jhong, J.-H., Lee, J., and Koo, J.-Y. (2017). Meta-analytic support vector machine for integrating multiple omics data. BioData Min. *10*, 2.
67. Ng, P.C., and Henikoff, S. (2003). SIFT: Predicting amino acid changes that affect protein function. Nucleic Acids Res. *31*, 3812–3814.
68. McLaren, W., Gil, L., Hunt, S.E., Riat, H.S., Ritchie, G.R.S., Thormann, A., Flicek, P., and Cunningham, F. (2016). The Ensembl Variant Effect Predictor. Genome Biol. *17*, 122.
69. Shukla, S.A., Rooney, M.S., Rajasagi, M., Tiao, G., Dixon, P.M., Lawrence, M.S., Stevens, J., Lane, W.J., Dellagatta, J.L., Steelman, S., et al. (2015). Comprehensive analysis of cancer-associated somatic mutations in class I HLA genes. Nat. Biotechnol. *33*, 1152–1158.
70. Hao, Y., Hao, S., Andersen-Nissen, E., Mauck, W.M., 3rd, Zheng, S., Butler, A., Lee, M.J., Wilk, A.J., Darby, C., Zager, M., et al. (2021). Integrated analysis of multimodal single-cell data. Cell *184*, 3573–3587.e29.

71. Benjamini, Y., and Hochberg, Y. (1995). Controlling the false discovery rate: A practical and powerful approach to multiple testing. J. R. Stat. Soc. *57*, 289–300.

72. Tate, J.G., Bamford, S., Jubb, H.C., Sondka, Z., Beare, D.M., Bindal, N., Boutselakis, H., Cole, C.G., Creatore, C., Dawson, E., et al. (2019). COSMIC: the Catalogue Of Somatic Mutations In Cancer. Nucleic Acids Res. *47*, D941–D947.

73. Karczewski, K.J., Francioli, L.C., Tiao, G., Cummings, B.B., Alföldi, J., Wang, Q., Collins, R.L., Laricchia, K.M., Ganna, A., Birnbaum, D.P., et al. (2020). The mutational constraint spectrum quantified from variation in 141,456 humans. Nature *581*, 434–443.

74. 1000 Genomes Project Consortium; Auton, A., Brooks, L.D., Durbin, R.M., Garrison, E.P., Kang, H.M., Korbel, J.O., Marchini, J.L., McCarthy, S., McVean, G.A., and Abecasis, G.R. (2015). A global reference for human genetic variation. Nature *526*, 68–74.

75. Wang, C., Huang, H., and Davis, M.M. (2022). Grouping T-Cell Antigen Receptors by Specificity. Methods Mol. Biol. *2574*, 291–307.

CellPress
OPEN ACCESS

STAR★METHODS

KEY RESOURCES TABLE

REAGENT or RESOURCE	SOURCE	IDENTIFIER
**Biological samples**		
Tumor tissue (Tissue-Tek O.C.T Compound embedded tissue or punches of formalin-fixed paraffin embedded tissue (FFPE))	Christensen et al.[16] Strandgaard et al.[1]	N/A
Frozen blood samples (leukocyte DNA was extracted from buffy coat, cell-free DNA was extracted from plasma)	Christensen et al.[16] Strandgaard et al.[1]	N/A
Fresh blood samples	This paper	N/A
Fresh bladder tumor biopsies	This paper	N/A
**Critical commercial assays**		
Puregene DNA purification kit	Gentra Systems	Cat#80234
QIAsymphony DSP DNA midi kit	QIAGEN	Cat#937255
QIAamp Circulating Nucleic Acid Kit	QIAGEN	Cat#61504
AmpliSeq™ TCR beta-SR Panel	Illumina	Cat#20031675
AmpliSeq™ Library PLUS (96 Reactions)	Illumina	Cat#20019102
AmpliSeq™ UD Indexes	Illumina	Cat#20019104
EasySep™ Human CD3 Positive Selection Kit II	STEMCELL	Cat#17851
EasySep™ Direct Human T cell Isolation Kit	STEMCELL	Cat#19661
Chromium Next GEM Single Cell 5′ Kit v2 (Dual Index)	10X Genomics	Cat#1000263/5
Chromium Single Cell V(D)J Amplification Kits, Human	10X Genomics	Cat#1000252
Chromium Next GEM Chip K Single Cell Kit	10X Genomics	Cat#1000286/7
Dual Index Kit TT Set A	10X Genomics	Cat#1000215
**Deposited data**		
TCR-seq data of tumor and blood (MIBC and NMIBC)	This paper	EGAS50000000940
RNAseq data from blood	This paper	EGAS50000000939
Single-cell paired RNAseq and TCR-seq of tumor and blood	This paper	EGAS50000000938
TCGA (germline and tumor WES; only stage I-III MIBC)	Robertson et al.[21]	https://portal.gdc.cancer.gov/legacy-archive
IMvigor210 (germline and tumor WES)	Mariathasan et al.[22]	EGAD00001004218
MSigDb	Subramanian et al.[46] Liberzon et al.[47]	https://www.gsea-msigdb.org/gsea/msigdb/index.jsp
VDJdb	Shugay et al.[48]	https://vdjdb.cdr3.net
**Software and algorithms**		
Custom scripts to create all figures	This paper	Zenodo: https://doi.org/10.5281/zenodo.14823577
blcfastq (v2.20.0.422)	Illumina	https://support.illumina.com/content/dam/illumina-support/documents/documentation/software_documentation/bcl2fastq/bcl2fastq_letterbooklet_15038058brpmi.pdf
MiXCR (v3.0.13)	Bolotin et al.[49,50]	https://mixcr.com/
Salmon	Patro et al.[51]	https://combine-lab.github.io/salmon/
R (v4.1.2)	R Core Team[52]	https://www.r-project.org/
DESeq2 (v.1.38.3)	Love et al.[53]	https://bioconductor.org/packages/release/bioc/html/DESeq2.html
EnsDb.Hsapiens.v86 (v.2.99.0)	Rainer[54]	https://bioconductor.org/packages/release/data/annotation/html/EnsDb.Hsapiens.v86.html
ReactomePA (v.1.42.0)	Yu et al.[55]	https://bioconductor.org/packages/release/bioc/html/ReactomePA.html
Kallisto (v.0.48.0)	Bray et al.[56]	https://github.com/pachterlab/kallisto

(*Continued on next page*)

Continued

REAGENT or RESOURCE	SOURCE	IDENTIFIER
EdgeR (v3.40.2)	Robinson et al.[57]	https://bioconductor.org/packages/release/bioc/html/edgeR.html
GSVA (v.1.46.0)	Hänzelmann et al.[58]	https://www.bioconductor.org/packages/release/bioc/html/GSVA.html
GATK (v4.4.0.0)	McKenna et al.[59]	https://github.com/broadinstitute/gatk
Mutect2 (from GATK)	Cibulskis et al.[60]	N/A
SnpEff (v4.3i)	Cingolani et al.[61]	https://pcingola.github.io/SnpEff/
TcellExTRECT	Bentham et al.[20]	https://github.com/McGranahanLab/TcellExTRECT
annovar (annotate_variation.pl, version from 2018 to 04-16)	Wang et al.[62]	https://annovar.openbioinformatics.org/
vcfR (v1.14.0)	Knaus et al.[63]	https://knausb.github.io/vcfR_documentation/
data.table (v1.14.8)	Dowle and Srinivasan[64]	https://cran.r-project.org/web/packages/data.table/vignettes/datatable-intro.html
tidyverse (v2.0.0)	Wikham et al.[65]	https://www.tidyverse.org/
MetaSVM	Kim et al.[66]	
SIFT	Ng et al.[67]	https://sift.bii.a-star.edu.sg/
VEP (v107)	McLaren et al.[68]	https://www.ensembl.org/info/docs/tools/vep/index.html
POLYSOLVER	Shukla et al.[69]	http://archive.broadinstitute.org/cancer/cga/polysolver
Kraken2	Wood et al.[28]	https://github.com/DerrickWood/kraken2/wiki
GLIPH2	Huang et al.[25]	http://50.255.35.37:8080/
Cell Ranger	10X Genomics	https://github.com/10XGenomics/cellranger
Seurat (v4.4.0)	Hao et al.[70]	https://satijalab.org/seurat/
CellTypist	Domínguez Conde et al.[30]	https://www.celltypist.org/

EXPERIMENTAL MODEL AND STUDY PARTICIPANT DETAILS

Human participants

National Scientific Ethical Committee granted permission to perform this project (#1706291; #1302183; #1708266). Written informed consent was obtained from all patients before inclusion in the study. The study's main cohort consists of 119 patients with localized MIBC, prospectively enrolled between 2013 and 2022. The 119 patients had a median age of 70 at inclusion (range 40–80) and most of the patients were males (95, based on gender; Table S2). Blood samples were collected at uniformly scheduled clinical visits during a two-year follow-up period. The patients with MIBC were recruited for a prospective study of ctDNA[16] based on having received neoadjuvant/first-line cisplatin-based chemotherapy followed by radical cystectomy. As part of these previously published studies,[16–18] a subset of the patients had been subjected to WES (n = 67) of tumor and blood samples and total RNA-seq (n = 44) of tumor samples. Follow-up data have been updated since the first publication and ctDNA has been reevaluated using WGS (n = 119).[19] Patients were categorized as metastatic if metastases were detected by computed tomography (CT) scan or other clinical follow-up after a cystectomy attempt (n = 31). Non-metastatic patients were disease-free with at least two years of follow-up (n = 79) after cystectomy. Nine patients had insufficient follow-up (< two years) or died within two years, and were excluded from all analyses comparing patients with and without metastatic disease. Three patients with non-successful cystectomy or death before the first CT scan were excluded from RFS curves. Pathological complete response was defined as T0,N0 after chemotherapy, while the non-invasive response was defined as T1,CIS,N0 or less. We performed TCR-seq on blood samples taken at diagnosis (before administration of neoadjuvant chemotherapy). A subset of patients was included for TCR-seq on tumor samples (n = 28) and longitudinal blood samples taken after chemotherapy, three weeks after cystectomy, and either when metastatic disease was detected or one year after cystectomy (n = 33). The samples were selected based on the availability of blood samples at the relevant time points and paired tumor WES data, ensuring a balanced representation between patients who developed metastasie and those who did not. Biochemical laboratory measurements of blood cell counts were obtained through patient journals, when available. The study's second cohort is a subset of 30 patients from a larger study cohort of 156 patients with NMIBC,[1] where the inclusion criteria was a minimum of 5 cycles of BCG immunotherapy. This study is retrospective, and the 30 patients were selected to be balanced between BCG responders and non-responders. Blood samples collected before and after BCG along with BCG-naive tumor samples from 30 patients were subjected to TCR-seq. The 30 patients had a median age of 70 at BCG induction (range 30–80) and most of the patients were males (23, based on gender; Table S2). Patients were categorized into early high-grade recurrence or progression (n = 15) if

high-grade urothelial carcinoma was detected within two years after the end of BCG induction treatment or if patients progressed to MIBC at any time during follow-up. Patients with late or no high-grade recurrence (n = 15) were free of high-grade tumors for at least two years after the end of BCG induction treatment. Blood and tumor WES data were available for all patients in the full cohort (n = 156).[1] For single-cell analysis, we included four patients undergoing cystectomy at the Department of Urology, Aarhus University Hospital, Denmark in May and June 2023. None of these patients were treated with chemotherapy prior to cystectomy. These patients had a median age of 72 (range 58–80) and all were males (based on gender).

METHOD DETAILS

DNA and RNA extraction

DNA was purified from frozen buffy coat samples on QIAsymphony SP using QIAsymphony DSP DNA Midi Kit (QIAGEN). Tumor DNA was purified from fresh-frozen tumors or formalin-fixed paraffin-embedded tumors using Gentra Puregene Tissue Kit (QIAGEN) or AllPrep DNA/RNA Kit (QIAGEN), respectively.

Blood buffy coat RNA was purified from frozen buffy coat samples using miRNeasy Mini Kit (QIAGEN). Approximately 150 mm^3 of frozen blood was cut out of a cryogenic tube using a scalpel and placed in a 2 mL Eppendorf tube. The cells were disrupted by mixing with 1.5 mL QIAzol Lysis Reagent before adding 140 μL chloroform. The remaining steps were performed according to protocol.

The quality of DNA and RNA was assessed using TapeStation4200 (Agilent) and the yield was determined using Qubit Fluorometric quantification (ThermoFisher Scientific) and DropSense96 (Trinean) of the DNA and RNA, respectively.

Library preparation and sequencing

AmpliSeq for Illumina TCR beta-SR panel was used to create TCR libraries for sequencing using 200 ng input DNA. The quality of TCR libraries was assessed using TapeStation4200 (Agilent) and Quibit Fluorometric quantification (ThermoFisher Scientific). The TCR libraries were paired-end sequenced on the Illumina NovaSeq6000 platform using SP and S1 flow cells (v1.5, 2x101 cycles), yielding an average of 42 million reads covering the CDR3β region (range 5–178 million).

Illumina Stranded Total RNA Prep with Ribo-Zero Plus kit was used to generate RNA libraries using 50 ng input RNA purified from buffy coat. The quality and yield of RNA libraries were assessed using TapeStation4200 (Agilent) and Quibit Fluorometric quantification (ThermoFisher Scientific). Sequencing was performed on the Illumina NovaSeq6000 platform using S2 flow cells (v1.5, 2x150 cycles), yielding an average of 305 million reads (range 101–357 million).

TCR-seq data analyses

The raw base call files were demultiplexed into FASTQ files using blcfastq (v2.20.0.422) from Illumina, allowing one mismatch in the index sequence. Subsequently, TCR clones were extracted from the data with MiXCR (v3.0.13)[49,50] using the analyze amplicon function with 5-end v-primers, 3-end j-primers, and adapters present. Subsequently, clones with less than 50 reads were filtered out. These extremely low-frequency clones were characterized by abnormal length and frequent frameshifts, indicating that they were mainly sequencing artifacts. To have one TCR representing each clone, we removed non-productive TCR protein sequences from further analysis. Clones were defined based on nucleotide sequence unless otherwise mentioned. The normalized Shannon diversity index was calculated using the equation:

$$Normalized\ Shannon\ diversity\ index = -\frac{1}{log\ (N)} \cdot \sum_{(N, i = 1)} p_i \cdot log\ (p_i)$$

Where N is the total amount of clones in a sample and p_i is the frequency of clone i. The fraction of hyper-expanded clones was quantified as the total frequency of clones above a frequency of 0.002. Overlapping TCR clones were determined based on the CDR3β nucleotide sequence. To reduce plot size, bubble plots of bulk blood TCR repertoires show top clones corresponding to 75% of the total repertoire frequency. Enrichment/depletion of clones relative to baseline was calculated using a binomial test, under the assumption of random clone sharing with the baseline repertoire. For any given clone in the binomial test, the probability corresponds to the frequency in the baseline sample. The number of trials corresponds to the amount of T cells surveyed in the paired sample. The number of unique TCRs was used as a lower-bound estimate for the number of T cells. The observed number corresponds to the frequency in the paired sample multiplied by the number of unique TCRs in the paired sample, rounded to the nearest integer. A T cell was considered enriched/depleted using a significance level of 0.05 after Bonferroni correction.

RNA-seq data analyses

Tumor RNA-seq was previously quantified using Salmon,[51] utilizing Gencode annotations (v.33) on GRCh38 as in Lindskrog et al.[18] Differential gene expression analysis was performed in R using DESeq2 (v.1.38.3),[53] which uses a Wald test. This analysis included 44 patients with MIBC, 21 with high TCR diversity, and 23 with low TCR diversity. Only protein-coding genes, excluding mitochondrial genes, were kept for analysis resulting in 18,665 genes from EnsDb.Hsapiens.v86 (v.2.99.0).[54] Genes with a count of less than 10 in at least 21 samples (smallest group) were excluded. The top 2000 most variable genes were selected based on standard deviation. P-values were adjusted for multiple testing using the false discovery rate (FDR),[71] and genes with an adjusted p-value below 0.25

were considered significant. Per category, the significantly upregulated genes (n = 153 for patients with low diversity, n = 39 for patients with high diversity) were used to calculate Reactome pathway enrichments using ReactomePA (v.1.42.0),[55] which estimates enrichments based on a Hypergeometric model.

Blood RNA-seq was quantified using Kallisto (v.0.48.0)[56] utilizing Gencode annotations (v.37) on GRCh38. Transcript counts were collapsed into counts at the gene level (Data S1). Gene expression count data were normalized using EdgeR's (v3.40.2)[57] Trimmed-Mean of M-values. We downloaded the gene set from Travaglini et al.[31] as a part of the C8 gene set collection from MSigDB[46,47] and conducted a Gene Set Variation Analysis using GSVA (v.1.46.0).[58] A GSVA score for naive and effector T cells was found by combining the "CD8 naive T cells" set with the "CD4 naive T cells" set, and the "CD8 effector memory T cells" set with the "CD4 effector memory T cells" set, respectively. We calculated the ratio of naive to effector T cells and compared patients with low and high TCR diversity.

WES data analyses

Tumor and buffy coat WES data from 67 patients in the MIBC cohort, previously produced, were reanalyzed using the GRCh38 reference genome. Fastq files were trimmed using cutadapt and mapped with bwa-mem using the GRCh38 genome assembly. Duplicate reads were marked using MarkDuplicates from GATK[59] and base quality scores were recalibrated (ApplyBQSR, GATK). Variants were called using Mutect2 and annotated using SnpEff (v4.3i).[61] Finally, variants with a frequency below 5% (VAF <5%), less than three alternate allele reads in the tumor, or a ROQ score below 30 (Phred-scaled probability that the variant alleles are not due to a read orientation artifact) were filtered out.

Additionally, 110 of the 156 patients in the NMIBC cohort had WES data captured with the Twist Human Core Exome Capture kit available. These samples were included for estimating T cell fractions. Reads were aligned and processed as described above. Of these 110 patients only 9 overlapped with the TCR analysis (total patients included = 131).

Based on germline WES data, we estimated blood T cell fractions using TcellExTRECT with default settings. For MIBC we used capture targets of TCRA genes from the SeqCapEZ MedExomeV1_hg19 capture kit. For NMIBC we estimated T cell fraction using the Twist human core exome capture targets.

For the tumor WES data, VCF-files were annotated using annovar (annotate_variation.pl, version from 2018 to 04-16),[62] vcfR (v1.14.0),[63] data.table (v1.14.8),[64] and tidyverse (v2.0.0).[65] Mutations were considered driver mutations if they were either 1) in a list of known driver mutations; 2) single nucleotide variants in tumor suppressor genes that were either predicted deleterious by MetaSVM[66] or SIFT,[67] or annotated as being a stop-gain or splice mutation; 3) single nucleotide variants in oncogenes present at least three times in COSMIC (v90; cancer.sanger.ac.uk)[72]; or 4) any given mutation annotated as being either a non-synonymous, a stop-gain or a splice mutation that is present at least ten times in COSMIC. For the ten most mutated genes in MIBC from TCGA,[21] we counted the number of patients with and without driver mutations, with high and low TCR diversity, respectively. For each gene, we tested the difference in the number of driver mutations between patients with high and low TCR diversity using a Fisher's Exact test.

CHIP was called based on WES data from buffy coat samples. Mutations were called using Mutect2 (GATK v4.4.0.0),[60] using GnomAD[73] variants as germline reference. A panel of normals was created from 25 tumor samples without CHIP mutations, found by running the pipeline on all tumor samples using a panel of normals generated from the 1000 genomes project.[74] Subsequently, variants were filtered using FilterMutectCalls, and variants with germline-like VAF were excluded (determined by non-significant deviance from VAF 0.5, binomial test). The remaining variants were annotated using VEP (v107).[68] CHIP mutations were defined using a previously published list of specific CHIP-associated mutations in 74 genes from Bick et al.[24] Bick et al. report that 75% of CHIP cases are caused by mutations in DNMT3A, TET2, and ASXL1, and mutations in PPM1D, JAK2, SF3B1, SRSF2, and TP53 caused the next 15%. Mutations in these genes can cause increased proliferation, leading to the expansion of hematopoietic cell clones. The full list of mutations is available in Table S2 in Bick et al.[24] CHIP mutations were defined by a list of CHIP-specific mutations in 74 genes used to annotate CHIP by Bick et al.[24] CHIP was called if a sample had at least one CHIP-related mutation with >2% VAF.

WGS data analyses

Plasma WGS data from 119 patients with MIBC were used to detect ctDNA as described in Nordentoft et al.[19] HLA type was called based on buffy coat WGS data from these 119 patients, using POLYSOLVER.[69]

To detect latent viruses we utilized plasma WGS data collected throughout treatment (total n = 973, median 8 samples per patient, median read count per patient = 950 million reads). We extracted unaligned reads from these samples and detected viral DNA using Kraken2.[28] A standard index created by the original authors was used as a reference (benlangmead.github.io/aws-indexes/k2). We used an index that contains DNA from other species to filter out reads originating from non-viral species, especially poor-quality human reads. EBV and CMV DNA detection was called for a patient if any of the patient's samples contained more than 1 read identified as CMV or EBV with a distinct minimizer above 10. Fisher's exact test was used to test for an association between the development of metastatic disease and CMV DNA detection.

Annotation of TCR sequences

To annotate TCR clones we started by clustering baseline MIBC CDR3β amino acid sequences based on local and global sequence similarity using GLIPH2[75] utilizing the provided TRB human v2.0 CD48 reference data and default parameters. WGS-based HLA type

was included in the analysis. The analysis was independent of clone size, as clone frequency was excluded. TCR sequences of abnormal length were not included in the analysis (≧ 25 residues). High confidence clusters were established by selecting clusters with at least 3 unique sequences in at least 3 patients, and with significant Vβ gene enrichment bias, resulting in 12,831 clusters with a total of 63,299 CDR3β sequences. To infer an antigen target for the clusters, we reclustered the CDR3β sequences together with a dataset of high-confidence CDR3β sequences with known antigen targets obtained from VDJdb.[48] Only CDR3β-antigen pairs with a VDJdb confidence score above zero were included in the study, resulting in 4437 unique pairs. Clusters that were found in both runs were analyzed further. Note that this two-step process ensures that the CDR3β-antigen pairs do not bias cluster formation. Clusters sharing sequence motifs with a CDR3β-antigen pair were considered to target that antigen. Clusters with multiple targets were defined to target the most common named antigen. Fisher's exact test was used to test for association between being included in a cluster with an inferred target and a clone being hyper-expanded, and a one-sided Fisher's exact test was used to test for inferred target enrichment among hyper-expanded clones.

Validation datasets

For TCGA we included all stage I-III patients with blood-derived normal samples (n = 262). The blood samples are chemotherapy-naive making them directly comparable to our baseline samples. Blood-derived normal WES bam files were downloaded from the GDC data portal and analyzed. Productive CDR3β sequences were extracted using MiXCR's analyze shotgun functionality. For diversity estimation, patients with more than two reads and at least two distinct clones were included in the analysis. Below this threshold, repertoires will always be perfectly diverse (normalized Shannon diversity index = 1), and thus diversity can not be estimated. Blood T cell fractions were estimated using TcellExTRECT using exons from the corresponding capture kit (Agilent custom V2 Exome) with a median coverage threshold set to five.

For the IMvigor210 dataset, PBMC WES fastq files were acquired from EGA (EGAD00001004218). Reads were aligned and processed as described previously. Blood T cell fractions were estimated using TcellExTRECT using covered targets from Agilent SureSelect All Exon v5 (S04380110) overlapping TCRA genes. Productive CDR3β sequences were extracted using MiXCR's analyze shotgun functionality. For diversity estimation, patients with more than two reads and at least two distinct clones were included in the analysis.

Single-cell analyses

Fresh tumor and blood samples were collected from the Department of Urology, Aarhus University Hospital, Denmark. The samples were placed on ice and transferred to the laboratory for immediate processing. The tumor samples were washed with PBS and dissected using scalpels before transferring them into a gentleMACS C tube containing 3 mL PBS with 2% FBS and 1 mM EDTA. The samples were further dissociated using the gentleMACS m_intestine_01 program. Cells were filtered through a 70 μm and a 50 μm mesh and then centrifuged at 1300 rpm for 5 min at 4°C. The pellets were resuspended in 1 mL PBS with 2% FBS and 1 mM EDTA and subsequently filtered through 40 μm flowmi filters. T cells were isolated from the cell suspensions using the EasySep Human CD3 Positive Selection Kit II (STEMCELL) according to protocol. T cells were isolated from 1mL of the blood samples using the EasySep Direct Human T cell Isolation Kit (STEMCELL) according to protocol. The concentration and viability of the samples were measured using Via1-Cassettes on a Nucleocounter NC-3000. Samples with a concentration below 700,000 cells/mL were centrifuged for 5 min at 250G at RT and resuspended in PBS to a concentration of 1,000,000 cells/mL. Samples with a concentration above 1,200,000 were diluted using PBS to a concentration of 1,000,000 cells/mL.

Chromium Next GEM Single Cell 5′ Reagent Kits v2 (Dual Index) from 10X Genomics was used for single cell sequencing and quality was measured under way using Tapestation4200, HS-D5000 (Agilent). The quantity of the final libraries was measured using Qubit Fluorometric quantification (ThermoFisher Scientific). All libraries were paired-end sequenced on the Illumina NovaSeq 6000 platform using SP and S1 flow cells (v1.5, 300 cycles).

The single-cell data were pre-processed (demultiplexed, reads aligned and filtered, barcodes and UMIs counted, and TCR clones determined) using Cell Ranger (10X Genomics). The data were filtered based on unique features and mapping to mitochondria genes, keeping cells with >200 and <2500 unique features and <10% mitochondria mapping using Seurat (v4.4.0).[70] Clones with a TCR β sequence were included for analyses. The cells were annotated using CellTypist[30] with the model Immune All Low which has high resolution and includes 98 different immune cell types. The cell type of a T cell clone was called using majority vote and only cells annotated as T cells were included in the analyses. Only the most abundant cell types are shown in the figures. All the remaining cell types are grouped as "others", including Trm cytotoxic T cells, Memory CD + cytotoxic T cells, Treg(diff), Follicular helper T cells, Type 1 helper T cells, CRTAM+ gamma-delta T cells, gamma-delta T cells, MAIT cells, Cycling T cells, CD8a/b(entry), and Double-positive thymocytes. Dimensionality reduction was done to construct UMAPs using Seurat (v4.4.0). In brief, each sample was normalized using NormalizeData, and the 2000 most variable genes were found using FindVariableFeatures. Then the samples were integrated using an integration pipeline: 2000 integration genes were identified across samples using SelectIntegrationFeatures, and integration anchors were calculated using FindIntegrationAnchors to align the datasets. The data was integrated using IntegrateData and subsequently scaled using ScaleData. A principal component analysis was run using RunPCA and the top 30 principal components were used for constructing UMAPs, with cells colored by sample type and T cell subtypes. We visualized canonical gene expression changes between the different subtypes using a heatmap.

QUANTIFICATION AND STATISTICAL ANALYSIS

All statistical analyses were performed using R (v4.1.2)[52] All pairwise comparisons were tested using a Wilcoxon Rank-Sum test, except when comparing paired data, where a Wilcoxon Signed Rank test was used. Unnormalized values were used for time-series laboratory count data. For boxplots, the center line represents the median, box limits represent upper and lower quartiles, and whiskers represent 1.5 times the interquartile range. For survival analyses, differences between Kaplan-Meier curves are tested using a likelihood ratio test, and the HR and 95% confidence interval are calculated using Cox proportional hazard regression. Cox proportional hazard regression of OS was used for univariate and multivariable analyses. Correlations were tested using Spearman's rank correlation. If other tests were used they are described in the relevant method section above. All statistical tests were two-sided unless otherwise stated. When relevant multiple testing correction was performed using FDR. P-values or FDR-adjusted *p*-values were considered significant when below 0.05 unless stated otherwise. Sample sizes (n) refers to the number of patients unless otherwise stated in the figure or figure legend.

Cell Reports Medicine, Volume *6*

Supplemental information

Low T cell diversity associates with poor outcome in bladder cancer: A comprehensive longitudinal analysis of the T cell receptor repertoire

Asbjørn Kjær, Nanna Kristjánsdóttir, Randi Istrup Juul, Iver Nordentoft, Karin Birkenkamp-Demtröder, Johanne Ahrenfeldt, Trine Strandgaard, Deema Radif, Darren Hodgson, Christopher Abbosh, Hugo J.W.L. Aerts, Mads Agerbæk, Jørgen Bjerggaard Jensen, Nicolai J. Birkbak, and Lars Dyrskjøt

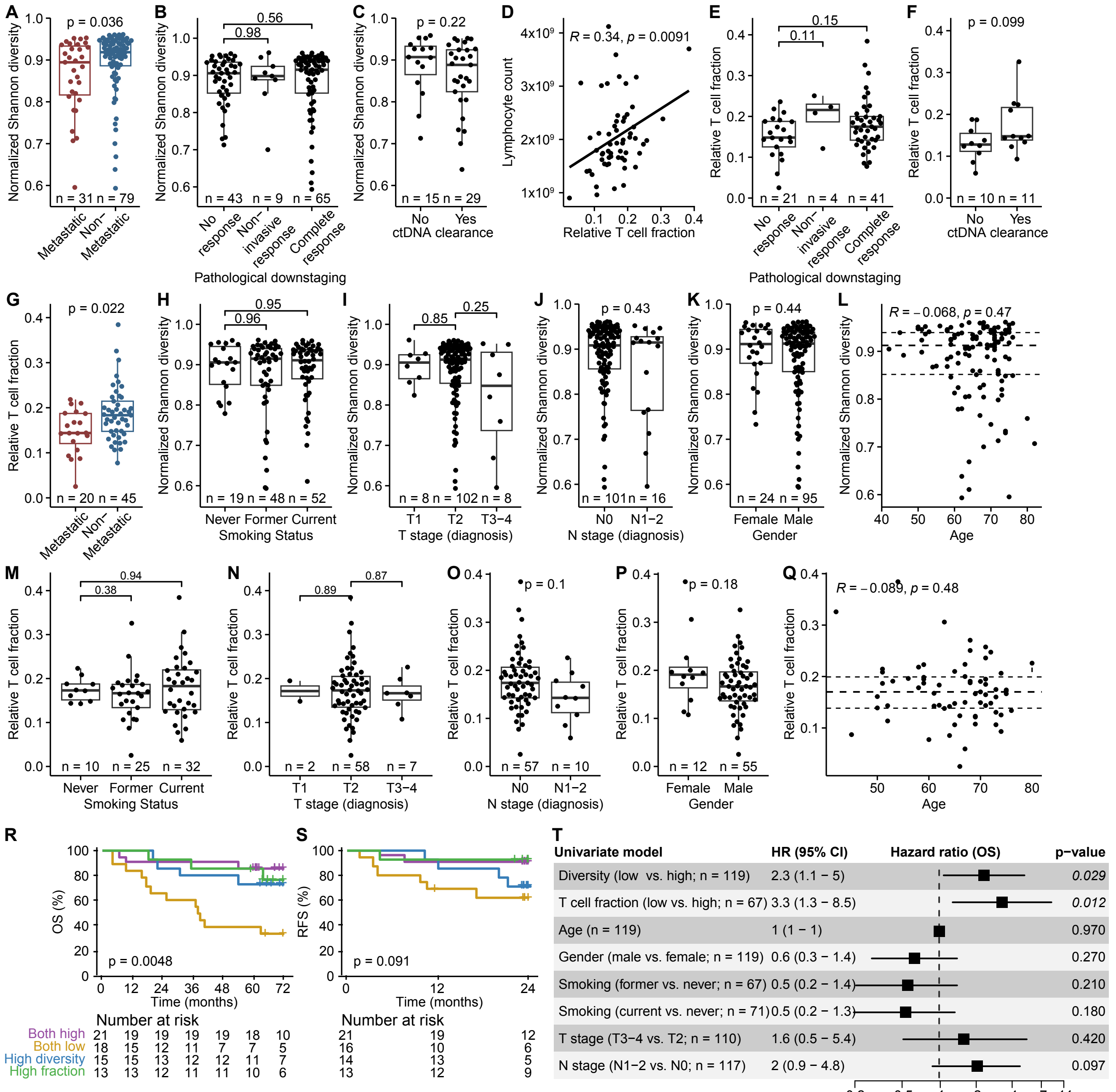

Figure S1. Baseline peripheral TCR features, relative T cell fraction, and clinical parameters in MIBC, related to Figure 2.
(A) Test of difference between normalized Shannon diversity and metastatic disease.
(B) Test of difference between normalized Shannon diversity and pathological downstaging.
(C) Test of difference between normalized Shannon diversity and ctDNA clearance.
(D) Spearman correlation of relative T cell fraction and lymphocyte count (n = 58).
(E) Test of difference between relative T cell fraction and pathological downstaging.
(F) Test of difference between relative T cell fraction and ctDNA clearance.
(G) Test of difference between relative T cell fraction and metastatic disease.
(H) Test of difference between normalized Shannon diversity and smoking status.
(I) Test of difference between normalized Shannon diversity and T stage.
(J) Test of difference between normalized Shannon diversity and N stage.
(K) Test of difference between normalized Shannon diversity and gender.
(L) Spearman correlation of normalized Shannon diversity and age (dashed lines indicate quartiles; n = 119)
(M) Test of difference between relative T cell fraction and smoking status.
(N) Test of difference between relative T cell fraction and T stage.
(O) Test of difference between relative T cell fraction and N stage.
(P) Test of difference between relative T cell fraction and gender.
(Q) Spearman correlation of relative T cell fraction and age (dashed lines indicate quartiles; n = 67).
(R) Survival analysis of OS for normalized Shannon diversity combined with relative T cell fraction, both split by median. High diversity indicates a high normalized Shannon diversity and low T cell fraction. High fraction indicates a high T cell fraction and low normalized Shannon diversity index.
(S) Survival analysis of RFS for normalized Shannon diversity combined with relative T cell fraction, both split by median. High diversity indicates a high normalized Shannon diversity and low T cell fraction. High fraction indicates a high T cell fraction and low normalized Shannon diversity index.
(T) Forest plot showing the HR for OS in univariate models of TCR diversity, T cell fraction, age, gender, smoking status, T stage, and N stage.
CI: confidence interval.

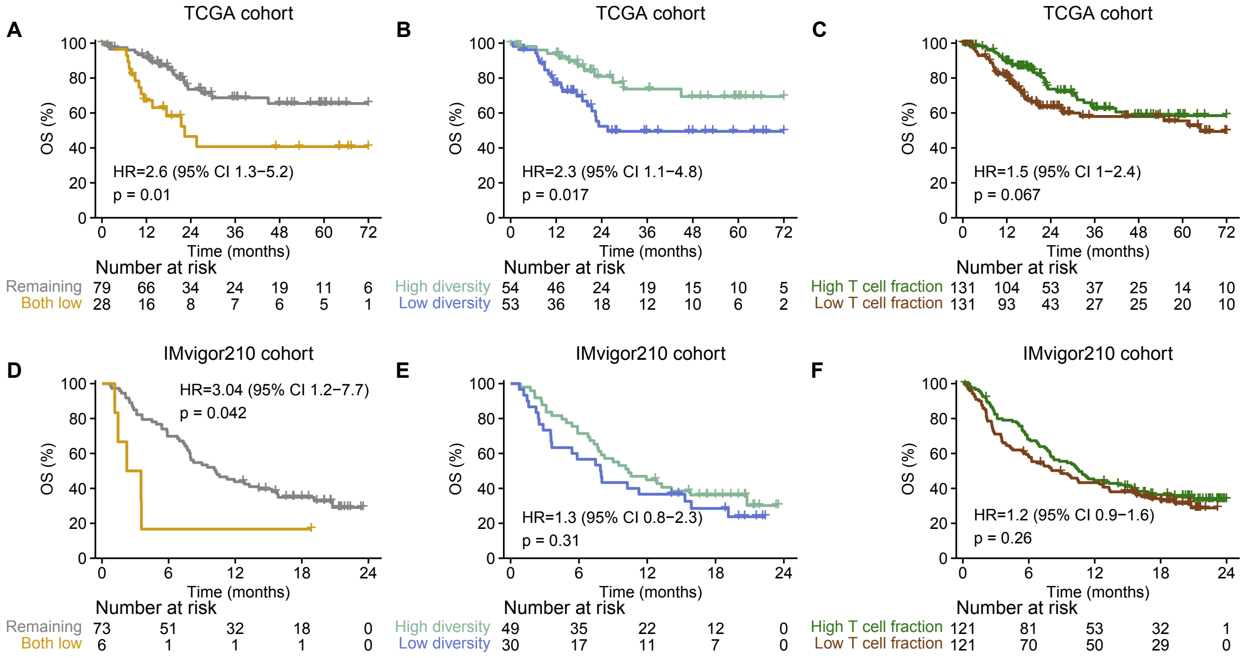

Figure S2. Analysis of TCR diversity and T cell fraction in independent cohorts, related to Figure 2.
(A-C) Survival analyses of stage I-III MIBC from TCGA (n = 262). The normalized Shannon diversity was determined by extracting TCR-beta sequences from germline WES samples. Only 107 patients had sufficient coverage of the CDR3 region to determine diversity. Survival analysis comparing OS for patients with low TCR diversity (median split) combined with low T cell fraction (median split) against the remaining patients (A), low and high (median split) TCR diversity (B), and low and high (median split) T cell fraction (C).
(D-F) Survival analyses of patients with metastatic bladder cancer from IMvigor210 (n = 242, n diversity = 79). Survival analysis comparing OS for patients with low TCR diversity (median split) combined with low T cell fraction (median split) against the remaining patients (D), low and high (median split) TCR diversity (E), and low and high (median split) T cell fraction (F).
CI: confidence interval.

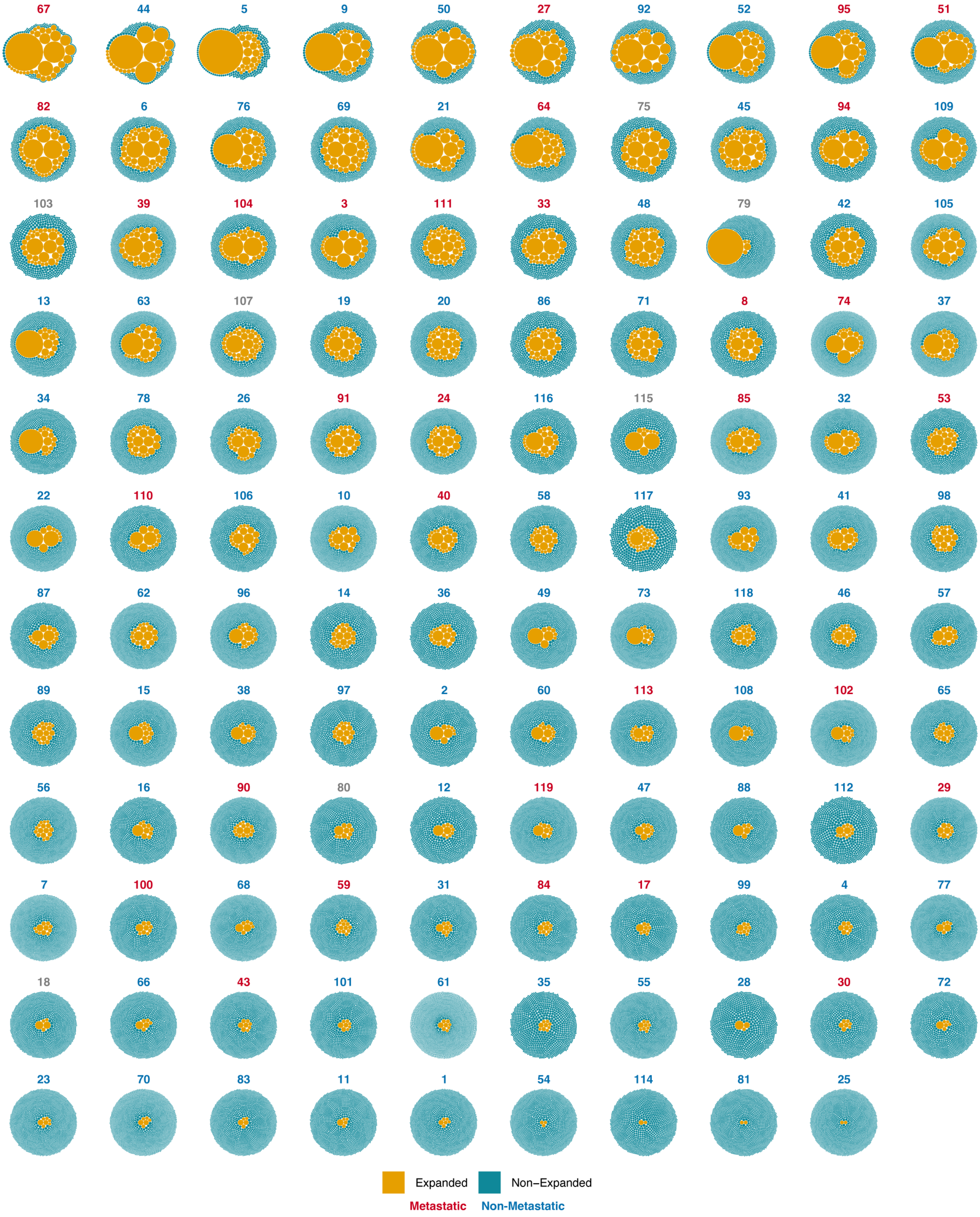

Figure S3. Bubble plots of baseline blood samples from the MIBC cohort, related to Figure 3.
One plot per patient. Each bubble represents a single TCR clone, with the size of the bubble representing the clone size. Colored by the size threshold for expansion. Patient labels colored by later development of metastatic disease.

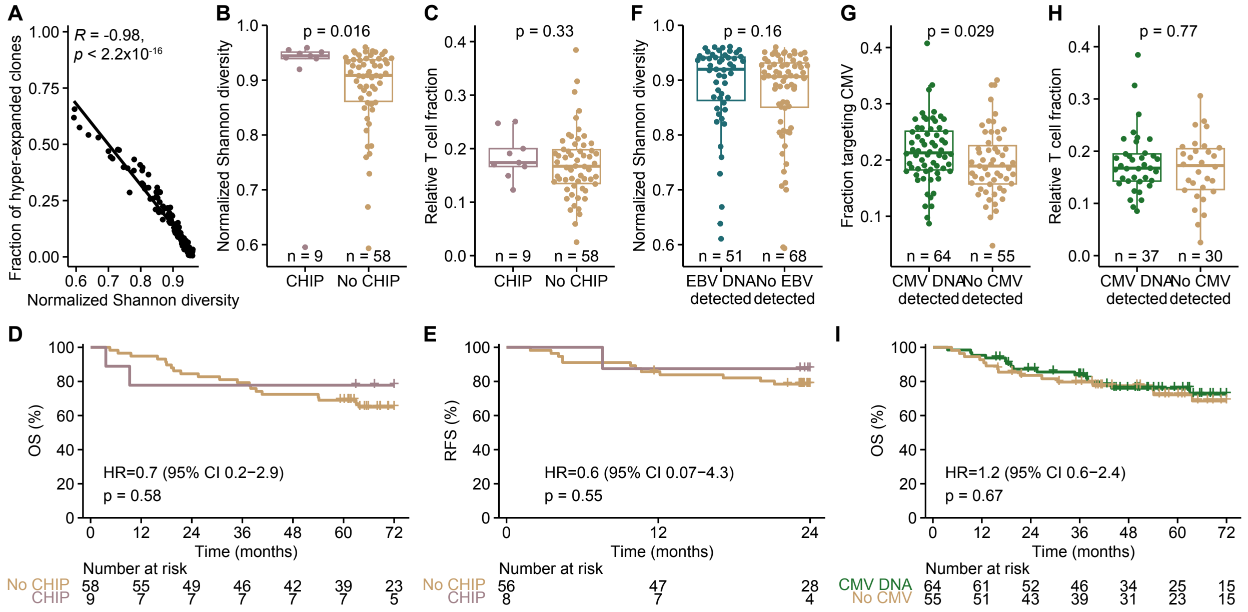

Figure S4. Analyses of CHIP and latent viral infections, related to Figure 3.

(A) Spearman correlation between the fraction of hyper-expanded clones and normalized Shannon diversity (n = 119).

(B-E) Analysis of CHIP in patients with MIBC. CHIP was called based on the detection of somatic, CHIP-associated mutations found in germline WES data.

(B) Test of difference between normalized Shannon diversity and CHIP.

(C) Test of difference between relative T cell fraction and CHIP.

(D) Survival analysis of OS for patients with and without detectable CHIP.

(E) Survival analysis of RFS for patients with and without detectable CHIP.

(F-I) Detection of CMV and EBV DNA in patients with MIBC. CMV and EBV DNA were detected based on WGS data from plasma samples using Kraken2.

(F) Test of difference between normalized Shannon diversity and detection of EBV DNA.

(G) Test of difference between fraction of TCRs targeting CMV and detection of CMV DNA.

(H) Test of difference between relative T cell fraction and detection of CMV DNA.

(I) Survival analysis of OS for patients with and without detectable CMV DNA.

CI: confidence interval.

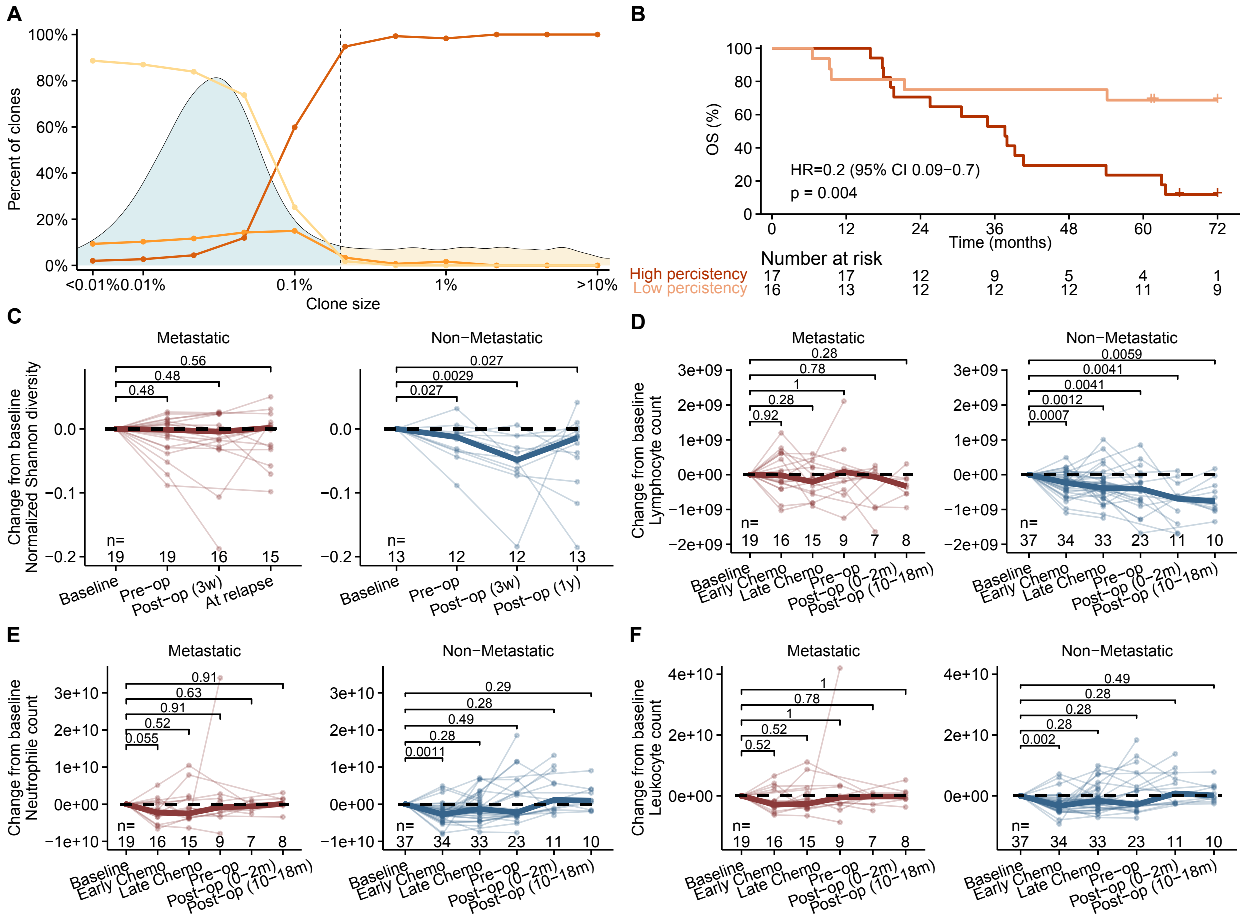

Figure S5. Analysis of persistency and blood lab measurements in MIBC, related to Figure 4.

(A) Association between clonal persistence and size of clones. Lines indicate the percentage of clones in each category at a specific size bin. Bins are uniformly distributed on log scale, x-axis placement indicates the end of the bin. All clones were concatenated for this analysis. The density plot shows the baseline size distribution of T cell clones (from Figure 3A). The dashed line as well as the blue and yellow colors indicate the threshold for defined hyper-expanded T cell clones.

(B) Survival analysis showing that high persistency (above median amounts of persistent and recurrent clones) is associated with OS.

(C-F) Change in immune landscape throughout treatment for patients with and without metastatic disease separately (two patients excluded due to incomplete follow-up); normalized Shannon diversity (C), lymphocyte counts (D), neutrophil counts (E), and leukocyte counts (F). Early chemo represents samples taken within one month of initiation, and late chemo represents the remaining samples. When multiple samples were available the average count was used. The thick lines indicate the median. Tests are based on the raw diversity measures or lab counts. P-values are FDR-adjusted.

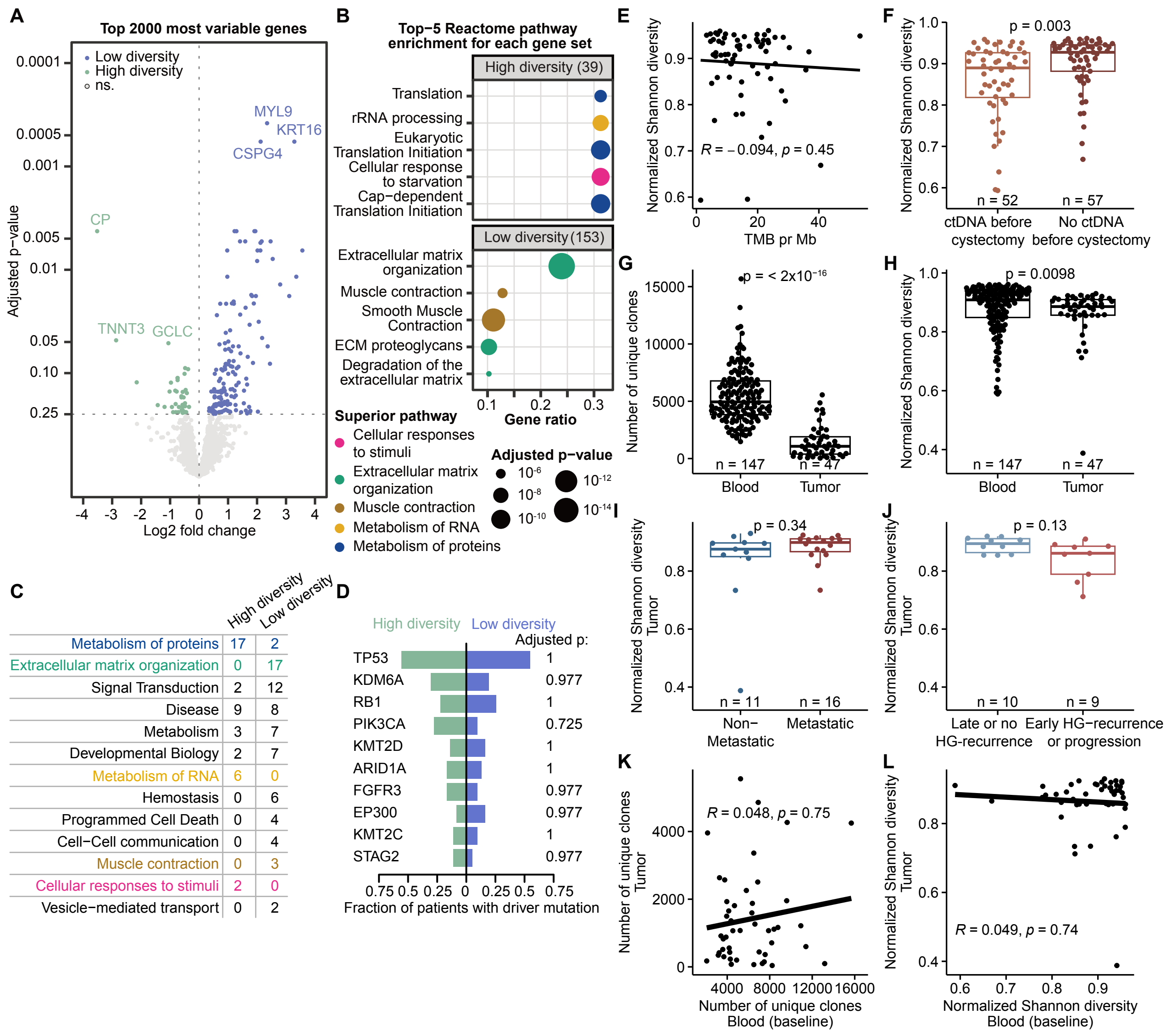

Figure S6. Association of peripheral TCR repertoires and tumor biology or tumor TCR repertoires, related to Figure 6.

(A) Differential gene expression analysis of the top 2000 most variable genes comparing patients with high and low TCR diversity for patients with MIBC (low diversity, n = 23; high diversity, n = 21).

(B) Reactome pathway enrichment analysis using significantly differentially expressed genes with higher expression in patients with high (n = 39) and low (n = 153) TCR diversity. The top five pathways for each group are shown.

(C-F) Analysis of peripheral TCR diversity relative to tumor biology.

(C) Summary of significant pathways from the Reactome pathway analysis in Figure 6B.

(D) Tree-plot showing the difference in the number of driver mutations between patients with high and low TCR diversity in ten bladder cancer driver genes (n, high diversity = 36; n, low diversity = 31).

(E) Spearman correlation of normalized Shannon diversity and TMB (n = 67).

(F) Test of difference between normalized Shannon diversity and ctDNA detection for patients with and without detectable levels of ctDNA before cystectomy.

(G-L) Joint analysis of paired tumor and baseline repertoires for patients with MIBC (n, blood = 119; n, tumor = 28) and NMIBC (n, blood = 28; n, tumor = 19).

(G) Test of difference between the number of unique clones in blood and tumor.

(H) Test of difference between the normalized Shannon diversity in blood and tumor.

(I) Test of difference between normalized Shannon diversity in the tumor and development of metastatic disease for patients with MIBC, with and without metastasis.

(J) Test of difference between normalized Shannon diversity in the tumor and recurrence status for patients with NMIBC, with late or no HG recurrence and early HG recurrence or progression.

(K) Spearman correlation between the number of unique clones in the tumor and in the blood (n = 47).

(L) Spearman correlation between the normalized Shannon diversity in the tumor and in the blood (n = 47).

HG: high-grade. TMB: tumor mutation burden.

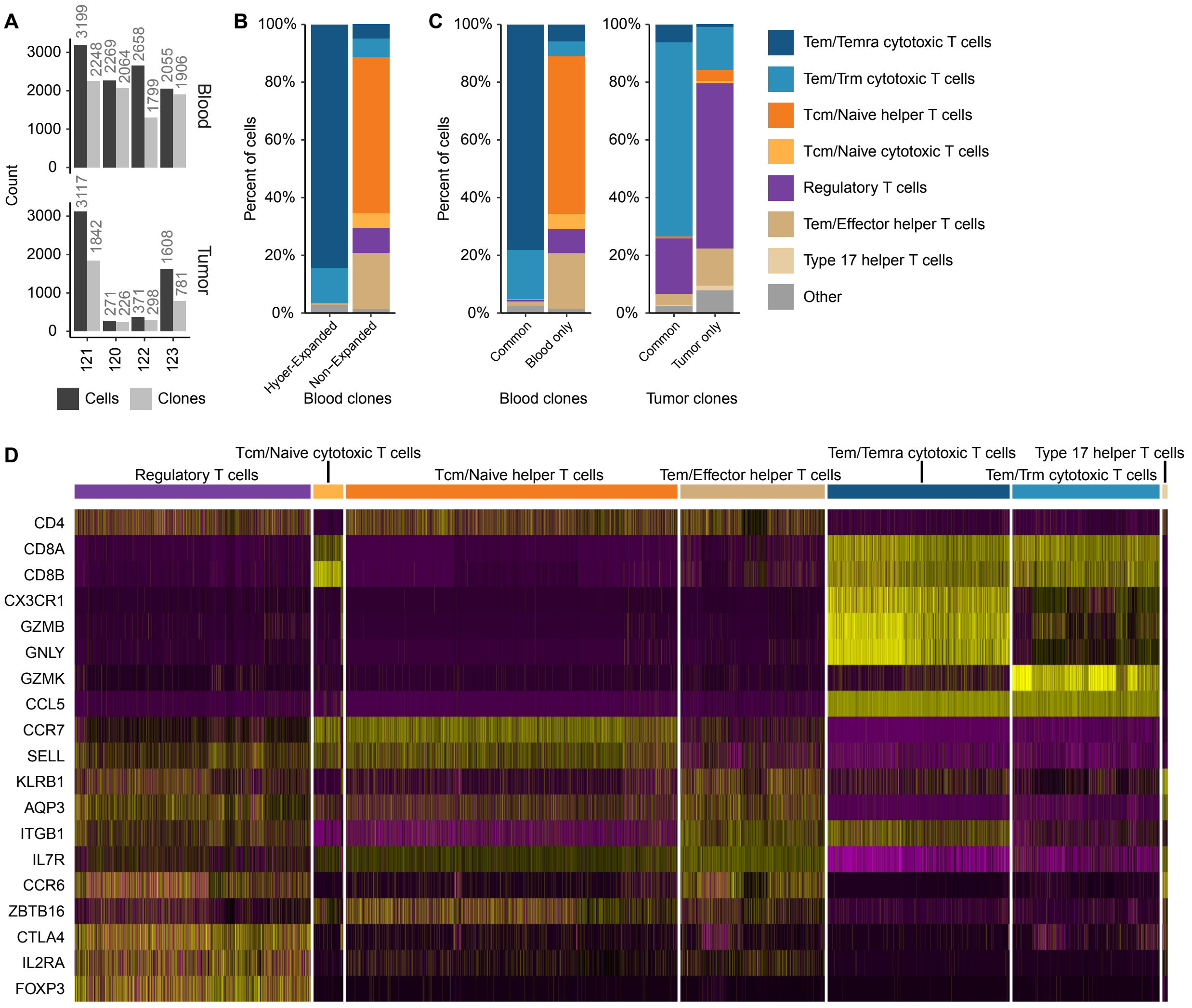

Figure S7. Single-cell analyses, related to Figure 7.

(A) Bar plots showing the number of T cells and unique clones for blood and tumor.

(B) Bar plots showing the distribution of cell types split by the T cell receptor being categorized as a hyper-expanded or non-expanded clone (n, cells with hyper-expanded clone = 2312; n, cells with non-expanded clone = 7869).

(C) Bar plots showing the distribution of cell types in blood and tumor cells, respectively, split by the TCR being either a common or a unique clone (n, cells with common clone in blood = 2390; n, cells with unique clone in blood = 7791; n, cells with common clone in tumor = 727; n, cells with unique clone in tumor = 4640).

(D) Heatmap showing gene expression from curated marked genes.

Tem: effector memory T cell. Temra: effector memory T cell reexpressing CD45RA. Trm: tissue-resident memory T cell. Tcm: central memory T cell.

Table S1 | Overview of analyzed samples, related to Figure 1

MIBC cohort (n = 119)

Patients from a prospective study cohort

Inclusion criteria: Neoadjuvant chemotherapy preceding radical cystectomy and available baseline blood sample

Data produced for this study		Previously produced data	
TCRseq	Baseline blood samples (n = 119)	WGS	Plasma samples (n = 119)
	Longitudinal blood samples (multiple time points, n = 33 patients)	WES	Blood and tumor samples (n = 67)
	Tumor samples (n = 30)	RNAseq	Tumor samples (n = 44)
RNAseq	Baseline blood samples (n = 8)		

Available laboratory blood measurements

Baseline and longitudinal measurements (multiple time points, n = 58 patients)

NMIBC cohort (n = 30)

Subset of patients from a retrospective study

Inclusion criteria: Treated with a minimum of 5 BCG instillations

Data produced for this study		Previously produced data (extended cohort)	
TCRseq	Baseline blood samples (n = 28)	WES	Blood and tumor samples (n = 110)
	After BCG blood samples (n = 28)		
	Tumor samples (n = 19)		

Single-cell cohort (n = 4)

New patients included for this study

Inclusion criteria: Chemotherapy-naive patients with bladder cancer undergoing a radical

Data produced for this study			
scRNAseq	Baseline blood samples (n = 4)		
	Tumor samples (n = 4)		

scRNAseq: single-cell RNAseq

Table S2 | Summary of patient characteristics, related to Figure 1

	MIBC (n = 119)	NMIBC (n = 30)
**Gender**		
Female	24 (20.2%)	7 (23.3%)
Male	95 (79.8%)	23 (76.7%)
**Age***		
Mean (SD)	70 (8)	70 (10)
Median [min, max]	70 [40, 80]	70 [50, 80]
**Smoking status**		
Current	52 (43.7%)	17 (56.6%)
Former	48 (40.3%)	11 (36.7%)
Never	19 (16.0%)	2 (6.7%)
**T stage at diagnosis**		
Ta	0 (0%)	18 (60.0%)
T1	8 (6.7%)	12 (40.0%)
T2	102 (85.8%)	0 (0%)
T3	1 (0.8%)	0 (0%)
T4a	4 (3.4%)	0 (0%)
T4b	3 (2.5%)	0 (0%)
Tx	1 (0.8%)	0 (0%)
**N stage at diagnosis**		
N0	101 (84.9%)	30 (100%)
N1	13 (10.9%)	0 (0%)
N2	3 (2.5%)	0 (0%)
Missing	2 (1.7%)	0 (0%)
**Recurrence****		
Recurrence	31 (26.0%)	15 (50.0%)
No recurrence	79 (66.4%)	15 (50.0%)
Follow-up < two years	9 (7.6%)	0 (0%)

* MIBC: age at diagnosis; NMIBC: age at BCG induction

** MIBC: detection of metastasis; NMIBC: early high-grade recurrence (< two years) or progression

SD: standard deviation
